# Tmod3 Phosphorylation Mediates AMPK-Dependent GLUT4 Plasma Membrane Insertion in Myoblasts

**DOI:** 10.3389/fendo.2021.653557

**Published:** 2021-04-20

**Authors:** Man Mohan Shrestha, Chun-Yan Lim, Xuezhi Bi, Robert C. Robinson, Weiping Han

**Affiliations:** ^1^ Laboratory of Metabolic Medicine, Singapore Bioimaging Consortium, Agency for Science, Technology and Research (A*STAR), Singapore, Singapore; ^2^ Bioprocessing Technology Institute, Agency for Science, Technology and Research (A*STAR), Singapore, Singapore; ^3^ Institute of Molecular and Cell Biology, Agency for Science, Technology and Research (A*STAR), Singapore, Singapore

**Keywords:** AMPK, GLUT4, myoblasts, Tmod3, translocation

## Abstract

Insulin and muscle contractions mediate glucose transporter 4 (GLUT4) translocation and insertion into the plasma membrane (PM) for glucose uptake in skeletal muscles. Muscle contraction results in AMPK activation, which promotes GLUT4 translocation and PM insertion. However, little is known regarding AMPK effectors that directly regulate GLUT4 translocation. We aim to identify novel AMPK effectors in the regulation of GLUT4 translocation. We performed biochemical, molecular biology and fluorescent microscopy imaging experiments using gain- and loss-of-function mutants of tropomodulin 3 (Tmod3). Here we report Tmod3, an actin filament capping protein, as a novel AMPK substrate and an essential mediator of AMPK-dependent GLUT4 translocation and glucose uptake in myoblasts. Furthermore, Tmod3 plays a key role in AMPK-induced F-actin remodeling and GLUT4 insertion into the PM. Our study defines Tmod3 as a key AMPK effector in the regulation of GLUT4 insertion into the PM and glucose uptake in muscle cells, and offers new mechanistic insights into the regulation of glucose homeostasis.

## Introduction

In addition to insulin stimulation, energy demanding conditions enhance glucose transport in skeletal muscle through GLUT4 redistribution to the plasma membrane (PM) through a distinct but separate signaling cascade other than insulin-signaling in skeletal muscle (1). When there is reduced energy supply in muscle cells under certain conditions like muscle contraction and deprivation of glucose or oxygen, glucose is replenished under the regulation of AMPK signaling (2).

AMPK acts as a sensor of cellular energy status in eukaryotic cells (3). It is activated by increased cellular [AMP]/[ATP] ratio caused by metabolic stresses, which accelerates ATP consumption and/or interferes with ATP production (4). AMPK activation in skeletal muscle correlates with enhanced glucose uptake (5, 6). AMPK activators, such as AICAR, adiponectin, IL-6, dinitrophenol and A-769662 promote GLUT4 trafficking to the cell surface and enhance glucose uptake in myotubes (7, 8).

Although AMPK-mediated GLUT4 translocation and insertion into the PM is a critical cellular process in the regulation of glucose uptake and glucose homeostasis, little is known at the molecular level regarding the downstream effectors of AMPK that play an essential role in the process, especially when compared with the well-studied Akt substrates in the regulation of GLUT4 trafficking mediated by the insulin signaling cascades (9–13). We have recently identified tropomodulin 3 (Tmod3), an actin filament capping protein, as an AKT2 substrate and characterized its role in the regulation of insulin-dependent GLUT4 translocation and glucose uptake in adipocytes (14). Phosphorylation of Tmod3 regulates insulin-induced cortical F-actin remodeling as an essential step for GLUT4 vesicle fusion with the PM. Furthermore, the interaction of Tmod3 with tropomyosin, Tpm3.1, is required for GLUT4 vesicle exocytosis and glucose uptake (14).

In this study, we report that Tmod3 is a novel effector of AMPK, and that Tmod3 phosphorylation by AMPK is essential for GLUT4 insertion into the PM and glucose uptake. Furthermore, AMPK-mediated Tmod3 phosphorylation regulates cortical F-actin remodeling and the binding of Tmod3 with monomeric G-actin is independent of the Tmod3: tropomyosin interaction. Taken together, our study provides a mechanistic link between AMPK signaling and cortical F-actin remodeling in GLUT4 insertion into the PM and glucose uptake in myoblasts.

## Materials and Methods

### Reagents

All the plasmids, antibodies and primers are listed in **Supplementary Tables 1**–**3** respectively. All the chemicals were purchased from Sigma-Aldrich unless otherwise stated.

### Cell Culture

L6 myoblasts ATCC (American Type Culture Collection) were cultured in α-MEM supplemented with 10% heat inactivated fetal bovine serum and 1% penicillin-streptomycin (10,000 U/mL) at 37°C and 5% CO_2_. HEK293T (ATCC) cells were cultured in high glucose DMEM supplemented with 10% fetal bovine serum and 1% penicillin-streptomycin.

### Lentivirus Packaging and Infection

All the lentiviruses were generated using third generation packaging system as per Biosafety Level 2 and institutional guidelines of Agency for Science, Technology and Research (A*STAR), Singapore. Lentiviruses were produced by co-transfection of HEK293T cells with lentiviral expression and packaging plasmids using the calcium phosphate transfection method as previously described (15, 16). Viral supernatant was collected after 48 hours of transfection and centrifuged at 1,000 x g for 5 minutes and filtered through 0.45 µm filter. The viruses were further concentrated by ultracentrifugation using SW41 swinging rotor in Beckman Coulter Optima-L-100 XP at 116,000 x g for 2 hours at 4°C. L6 myoblasts were infected with titrated viruses in the presence of 8 µg/mL of polybrene (Hexadimethrine bromide, Cat. No: 107689-10G, SIGMA-ALDRICH). The growth medium was replaced with normal growth medium after 24 hours of infection.

### Protein Purification

Tmod3-WT and its variants were purified as described previously with brief modifications (14).

#### Preparation of FLAG-Tagged Proteins From HEK293T Cells

FLAG tagged proteins were expressed in HEK293T cells and the cells were lysed in TNET buffer (50 mM Tris-HCl, pH 7.5, 150 mM NaCl, 10 mM NaF, 1 mM EDTA, 1% Triton X-100) supplemented with 2 mM Na_3_VO_4_ and 1 mM PMSF and 1X protease inhibitor cocktail tablet (Roche). The cleared supernatant was purified using anti-FLAG M2 Affinity gel (Sigma-Aldrich). Proteins were subsequently eluted with 3XFLAG peptide (Sigma-Aldrich) in TBS buffer (50 mM Tris-HCl, pH 7.5, 150 mM NaCl). Total protein concentrations in the samples were measured using Bradford assay.

#### Recombinant GST-Fusion Protein Purification

Full length mouse Tmod3-WT (Wild Type) and mutants were inserted in-frame in the linker region of pGEX-KG vector in order to encode fusion proteins with GST (Glutathione S-transferase) in the N-terminus. GST fusion Tmod3-WT and mutants were expressed in BL21 *Escherichia coli* and induced with 1 mM isopropyl-β-D-thiogalactopyranoside for protein production. Bacteria were harvested when OD_600_ of the bacterial growth reached approximately 1.00. The lysates were harvested and purified using standard protein purification protocol. Briefly, bacterial pellet was re-suspended in Buffer A containing 20 mM Tris-HCl, pH 8.0, 0.1 M NaCl, 1 mM DTT (dithiothreitol), 1 mM PMSF and 1X protease inhibitor cocktail tablet and incubated with 0.25 mg/ml lysozyme on ice for 30 min. The bacteria lysates were sonicated on ice and were treated with 0.5% Triton X-100 and rocked at 4°C for 15 minutes. The lysates were then cleared by centrifugation at 16,000 x g, at 4°C for 30 minutes. Glutathione-Sepharose 4B beads (GE Healthcare) were equilibrated in Buffer A containing 0.1% Triton X-100. The pre-equilibrated beads were incubated with clear supernatant for 1 hour at 4°C. The glutathione beads bound GST-fusion proteins were eluted by thrombin cleavage (GE Healthcare) in TTB buffer (20 mM Tris-Cl, pH 8.0, 2.5 mM CaCl_2_, 150 mM NaCl). Thrombin was removed using benzamidine-Sepharose beads (GE Healthcare) and thrombin free proteins were further concentrated using centrifugal filters (Millipore). Protein concentrations were measured using Bradford assay.

### Detection of Phospho-Ser25 of Tmod3 by LC-MS/MS

#### In-Gel Digestion and Nano-Electrospray Ionization-Tandem Mass Spectrometry

Phosphorylation of Ser25 of Tmod3 was detected by LC-MS/MS as described previously with brief modifications (17). Briefly, FLAG-tagged Tmod3 proteins were purified from HEK293T transiently co-expressing FLAG-Tmod3 and constitutively active form of AMPK (Myc-AMPK-CA) using anti-FLAG M2 Affinity gel. Proteins were eluted in 2x Laemmli buffer, heated at 95°C for 10 minutes and separated using 12% precast gels (Bio-Rad). The gel was stained with 0.1% Coomassie Brilliant Blue R250. Gel bands around 40 kDa which correspond to Tmod3 were excised, de-stained, de-hydrated with acetonitrile, treated with 25 mM DTT in 50 mM ammonium bicarbonate at 56°C for 25 minutes and alkylated in 55 mM iodoacetate for 30 minutes in the dark at room temperature. Samples were then incubated with 1 μg trypsin for 16 hours at 37°C. Then, the gel plugs were digested with 10 ng/μl mass spectrometry grade trypsin gold (Promega Gema) in 25 mM ammonium bicarbonate overnight at 37°C. Peptide mixtures were extracted with 20 mM ammonium bicarbonate, then with 50% (v/v) acetonitrile, 5% (v/v) formic acid in H_2_O. The pooled peptides, thus evaporated to dryness using SpeedVac, were dissolved in 5 μl of 2% (v/v) methanol and 1% (v/v) formic acid with 50mM citric acid for enhancing the detection by LC-ESI-MS/MS.

Mass spectrometry (MS) analysis was performed on a LTQ-Orbitrap Velos Pro ETD Mass Spectrometer (Thermo Fisher Scientific, San Jose, CA) that was equipped with nanoACQUITY UPLC system (Waters Milford, MA), Xcalibur 2.2 SP1.48 and LTQ Tune Plus 2.7 instrument control. 5 μl of peptides were desalted for 5 minutes with 0.1% (v/v) formic acid in water and 1% (v/v) acetonitrile in mobile phase at the rate of 8μL per minute. Nano-reverse-phase liquid chromatography, in nanoACQUITY UPLC BEH 130Å 1.7 μm C18 column, 75 μm x 200 mm, was used to separate desalted peptides keeping the flow rate of 0.3 μl/min at 35°C. Nanospray in positive ion mode at 1.8 kV was used for ionization. Data-dependent scanning tandem mass spectrometry was used to obtain spectra with one full MS scan from 300 to 1,800 m/z at resolution of 60,000, followed by HCD Orbitrap tandem MS scans of ten most intensive peptide ions with normalized collision energy of 35 v at a resolution of 15,000.

#### Mass Spectrometry Data Analysis

SequestHT and PhosphoRS3.0 in Protein Discoverer 1.4 SP1 software (ThermoScientific) were used to analyze tandem mass spectrometry data. MS raw data were also analyzed by database search using UniProtKB/Swiss-Prot, and annotation of the phospho-peptide tandem MS/MS using PEAKS studio 7.0 software (Bioinformatics Solutions Inc.) ± 5 ppm and ± 0.2 Da of the peptide and fragment ion mass tolerances were used respectively. Carbamido-methylation of cysteine was included as a fixed modification, oxidation of methionine, phosphorylation of serine, threonine and tyrosine were selected as dynamic and variable modification. Two missed cleavages were allowed for searching the data.

### *In Vitro* GST Pull-Down Assay

The glutathione beads bound GST-fusion proteins were equilibrated with TNET buffer. The beads and cell lysates containing 2 mg proteins were rocked at 4°C for overnight. The beads were washed with TNET buffer and eluted using 2X Laemmli sample buffer. The samples were subjected to SDS-PAGE and Western blot analysis.

### *In Vitro* Protein Kinase Assay

*In vitro* kinase assays were performed on purified FLAG-tagged Tmod3 WT and mutant proteins. Briefly, 1 μg of FLAG-​Tmod3 and 1 μg recombinant AMPK (α1/β1/γ2) (Sigma-Aldrich) were incubated in 20 μl of volume reaction in kinase buffer (25 mM ​Tris-HCl, pH 7.5, 5 mM ​β-glycerophosphate, 2 mM ​DTT, 0.1 mM ​Na_3_VO_4_, 10 mM ​MgCl_2_) containing 0.2 mM ​ATP. The kinase reaction was incubated at 30°C for 30 minutes and boiled in 30 μl 2X Laemmli sample buffer and separated by SDS-PAGE. Phosphorylation signals were detected using anti-phospho-serine antibody. For autoradiography, the sample mixtures were incubated in the kinase buffer with 10 μCi [^32^P] γ-ATP (PerkinElmer, SG) for 30 minutes at 30°C and subjected to SDS–PAGE and autoradiography.

### Radioactive Glucose Uptake Assay

2-Deoxyglucose uptake measurements were carried out as described previously (18) with some modifications. Briefly, L6 myoctyes were deprived of serum for 2 hours. Following all stimulations and inhibitions cell monolayers were washed thrice with KRPH (Krebs-Ringer Phosphate – Hepes, pH 7.4) buffer (19) and any remaining liquid was aspirated. Cells were then incubated for 5 minutes in KRPH buffer containing 10 μmol/l unlabeled 2-deoxyglucose and 10 μmol/l [^3^H]-2-deoxyglucose (1 μCi/ml) in the absence of AICAR and inhibitors, unless otherwise indicated. The reaction was terminated by washing thrice with cold PBS. The radioactivity was determined by lysing the cells with 0.05N NaOH, followed by liquid scintillation counting using PerkinElmer counter. Total protein was determined by the Bradford method. Nonspecific uptake was determined in the presence of 10 μmol/l cytochalasin B and subtracted from all experimental values. The glucose uptake values are expressed as “pmol radioactive 2-deoxyglucose taken up per minute and per mg protein.”

### Fluorescence Microscopy

Co-localization studies were carried out as described previously (14, 20). Briefly, L6 myoblasts plated on 0.1% gelatin-coated cover-slips were serum starved for 2 hours before AICAR treatment. Cells were fixed with 4% paraformaldehyde of pH 7.4, washed with ice cold PBS and blocked with PBS containing 5% goat serum (Cat. No: 16210072, GIBCO Life Technologies) and 0.02% sodium-azide for at least 1hour. Cells were then permeabilized with 0.01% Triton-X 100 in PBS and stained with relevant antibodies followed by Alexa Fluor secondary antibodies accordingly. The cover-slips were mounted with DAKO mounting medium (DAKO) after washing with PBS. Optical sections of each samples were taken through sequential scans at relevant wavelengths (488 nm and 561 nm) using a Nikon A1R-A1 confocal laser microscope system with X100 NA/1.40 CFI Plan APO VC oil-immersion objective. TIRFM setup was based on Nikon Eclipse-Ti inverted microscope with two EMCCD cameras (1,002 X 1,002 pixels, 8 X 8 μm, 14-bit, Andor iXon^EM^+885; Andor Technologies) capable of capturing two channels (TIRF laser 488 nm; TIRF laser 561 nm) simultaneously. Both TIRF and epifluorescence images were captured using X100 NA/1.49 APO TIRF oil-immersion objective. Immersion oil (ND=1.515, Nikon) was used to bridge the optical contact between the cover-slip and the objective. The penetration depth of the evanescent field is estimated to be ~200 nm. Images were acquired with no binning; at 13 mHz, the readout rate with average exposure times vary between 100 and 300 ms.

### Myc-​GLUT4-mCherry Translocation

For GLUT4 translocation studies, L6 myoblasts were transduced with lentivirus encoding shRNA against Tmod3 or Tmod3 mutants. After the AICAR treatment the cells were fixed and blocked in non-permeabilized condition. Then the cells were incubated with primary mouse anti-Myc Ab (9E10) for overnight and with Alexa Fluor 488-conjugated anti-mouse secondary antibody for 1 hour at 4°C. Mounted samples were subjected to confocal imaging at single centrally located plane. Nikon Element software was used for quantitative measurements of GLUT4 vesicle translocation. Briefly, the entire ventral surface of each cells expressing Myc-​GLUT4-mCherry was selected for measurement of fluorescence intensity of both mCherry and Myc-signals after removal of background fluorescence. The ratio of cell surface to total GLUT4 was quantified by detecting cell surface GLUT4 through anti-Myc fluorescence immuno-labelling and total GLUT4 through mCherry fluorescence in non-permeabilized L6 cells. Data in each group were normalized and expressed as a percentage of AICAR-treated control cells. TIRF/epifluorescence analysis for fixed cells was performed (21, 22). Under non-permeabilized conditions, the ratio of TIRF-Myc/Epi-mCherry was acquired by dividing the fluorescence intensity of TIRF-Myc signal for each cell by that of the Epi-fluorescence GLUT4-mCherry. Data in each group were averaged and the ratio of TIRF-Myc/Epi-mCherry in control group was set as 1 for group comparison.

### TIRFM-based F-Actin Remodeling and Analysis

TIRF based F-actin remodeling and analyses were done as described previously (14). L6 myoblasts expressing Lifeact-tdTomato were serum starved for 2hours and imaged using TIRFM with Perfect Focus System. AICAR (2 mM) was added at zero time point and TIRF images were taken over 30 minutes with an interval of 15 seconds and 121 total readings. Two independent ways of measurement were used to analyze the F-actin remodeling. (1) Measurement of TIRF-Lifeact-tdTomato on the cell periphery: this analysis was particularly focused on the AICAR-stimulated build-up or enrichment of cortical F-actin at the cell periphery. To quantify, multiple boxes of the region of interest [L X W: 5 X 5, 25 μm^2^ per region of interest (ROI)] spanning across the boundary of cell periphery were used to measure the fluorescence intensities of RAW images over time. In order to take into account the membrane reorganization in the time course, each ROI was selected to include a portion of the cell periphery and partial background. TIRF intensities of all ROIs were measured over time, all the readings were normalized to the intensity measured at zero time point after removing the background fluorescence, and the averaged values were plotted against the time so as to indicate the time course of F-actin remodeling under AICAR stimulation. This method selectively estimated the extent of cortical F-actin rearrangements on the cell periphery without considering the changes in intracellular ventral region. Events of membrane remodeling could be distinguished from the events of thickening of cortical F-actin. (2) Measurement of the TIRF-Lifeact-tdTomato on the ventral regions of the cell and away from the cell edge: ventral regions of each individual cell, excluding the peripheral regions of the cell were manually selected in Nikon Element software and subjected to time course analysis as described in (1) Measurement of TIRF-Lifeact-tdTomato on the cell periphery. The extent of ventral actin polymerization was assessed over time without taking into account the changes in membrane ruffling activity and peripheral F-actin structures.

### Statistical Analysis

Data were expressed as means ± SEM unless otherwise stated. Statistical analyses were performed using ANOVA. The levels of statistical significance were presented as asterisks and defined in each figure legend together with the name of the statistical test accordingly. The graphical data were analyzed using Microsoft Excel 2010 and Prism 7.0 (GraphPad, San Diego, CA).

## Results

### AMPK Regulates GLUT4 Translocation to the Cell Surface and Glucose Uptake in L6 Myoblasts

**Figure 1:**
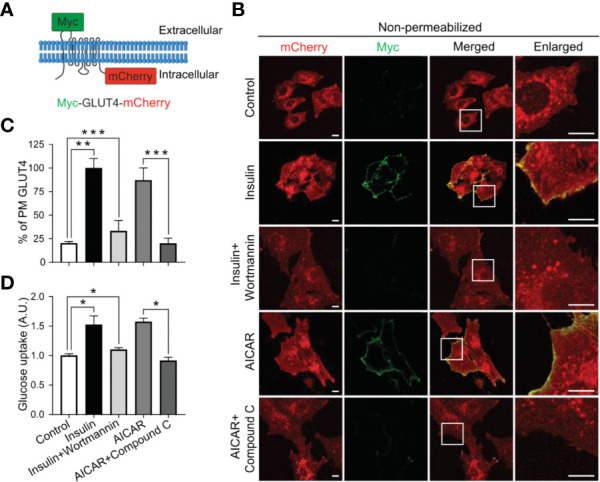
AMPK regulates GLUT4 translocation to the PM and glucose uptake in L6 myoblasts. **(A)** GLUT4 chimeric protein, Myc-GLUT4-mCherry, for the detection of GLUT4 translocation and insertion into the PM. **(B, C)** L6 myoblasts stably expressing Myc-GLUT4-mCherry were serum starved for 2 hours and treated with DMSO as control, insulin (100 nM) or insulin along with wortmannin (200 nM) for 20 minutes, AICAR (2 mM) or AICAR along with Compound C (CC) (10 µM) for 30 minutes. In fixed and non-permeabilized cells, cell surface Myc-GLUT4-mCherry signals were detected with anti-Myc mouse monoclonal antibody followed by Alexa Fluor-488 goat anti-mouse secondary antibody. The samples were subjected to confocal microscopic imaging. Images presented are from a single centrally located plane. Intensity of Myc at PM was quantified against intensity of total mCherry in the whole cell. Data in each group were normalized and expressed as a percentage of Insulin treated cells. Data are presented as mean ± SEM of about 100 cells in each group from three independent experiments (ANOVA with Dunnett’s multiple comparison test). ^**^p<0.01 and ^***^p<0.001. Scale bar: 10 μm. **(D)** Impaired [^3^H]-2-Deoxyglucose uptake in L6 myoblasts. After serum starvation for 2 hours, L6 myoblasts were treated with DMSO as control, insulin (100 nM), insulin plus wortmannin (200 nM) for 20 minutes, AICAR (2 mM) or AICAR plus Compound C (CC) (10 µM) for 30 minutes. Data are presented as mean ± SEM of three independent experiments (ANOVA with Dunnett’s multiple comparison test). ^*^p<0.05.

To investigate AMPK regulation of GLUT4 translocation and glucose uptake in myoblasts, we stably expressed a chimeric GLUT4 protein that includes a Myc epitope at the first exofacial loop and a fluorescent protein mCherry at the C-terminus (**Figure 1A**) in L6 myoblasts. GLUT4 translocation to the cell surface includes at least two steps: first, GLUT4 trafficking from the intracellular space to near the PM; and second, GLUT4 insertion into the PM, which results in the surface exposure of its exofacial loops. The chimeric protein allows tracking of GLUT4 localization, thus its trafficking to the cell periphery by mCherry fluorescence, and monitoring of GLUT4 insertion into the PM by antibody labelling of surface-exposed Myc at the first exofacial loop under non-permeabilized condition (14). Similar to insulin signaling activation, significant increase in GLUT4 translocation to the PM and surface exposure was observed upon AMPK activation, along with increased glucose uptake in both L6 myoblasts and myotubes (**Figures 1B–D** and **Supplementary Figure 1A**). Conversely, Compound C, an AMPK inhibitor, reduced both GLUT4 translocation and glucose uptake, as wortmannin did for insulin signaling (**Figures 1B–D** and **Supplementary Figure 1A**). These results are consistent with an established function of AMPK in the regulation of GLUT4 translocation to the PM and glucose uptake in L6 myoblasts, and demonstrate an experimental setup to allow examination of AMPK regulation of distinct steps of GLUT4 translocation.

### Tmod3 Phosphorylation by AMPK Is Essential for AMPK-Mediated GLUT4 Translocation to the PM and Glucose Uptake

Tropomodulins are actin filament capping proteins that regulates the length of actin filaments in muscle and non-muscle cells (23). All four Tmod isoforms were detected in L6 myoblasts, however, Tmod3 was found to be predominantly expressed (**Supplementary Figure 1B**). Tmod3 expression was not altered with the differentiation of L6 myoblasts into myotubes (**Supplementary Figure 1C**). Tmod3 has been found to be localized mainly in the cytoplasm but sparsely in the cortex in the maturing oocytes (24). Tmod3 was reported to be localized preferentially at the F-actin rich structures in human microvascular endothelial cells (25). However, Tmod3 does not localize prominently to perpendicular F-actin bundles of the cell body and diffused distribution of Tmod3 is observed in the cytoplasm which is likely due to fixation of a portion of the soluble Tmod3-pool (25). Consistently, we found that Tmod3 was associated with phalloidin-labeled cortical F-actin and co-localized with GLUT4 at cell periphery under AMPK activation conditions (**Supplementary Figures 2A–F**). Moreover, a subset of Tmod3 was co-localized with phospho-AMPK under AMPK activation (**Supplementary Figures 3A–C**). Tmod3 KD L6 myoblasts were generated using shRNA against Tmod3 and control shRNA to study the association of Tmod3 with phospho-AMPK (**Supplementary Figure 1D**). Not surprisingly, the co-localization of Tmod3 with the phospho-AMPK was significantly reduced while the level of phospho-AMPK remained unaltered in Tmod3 KD L6 myoblasts (**Supplementary Figures 3D–F**) indicating that the silencing of Tmod3 has no role in AMPK phosphorylation. These data suggest a potential role of Tmod3 in regulating AMPK-mediated GLUT4 translocation and glucose transport through cortical F-actin remodeling.

**Figure 2:**
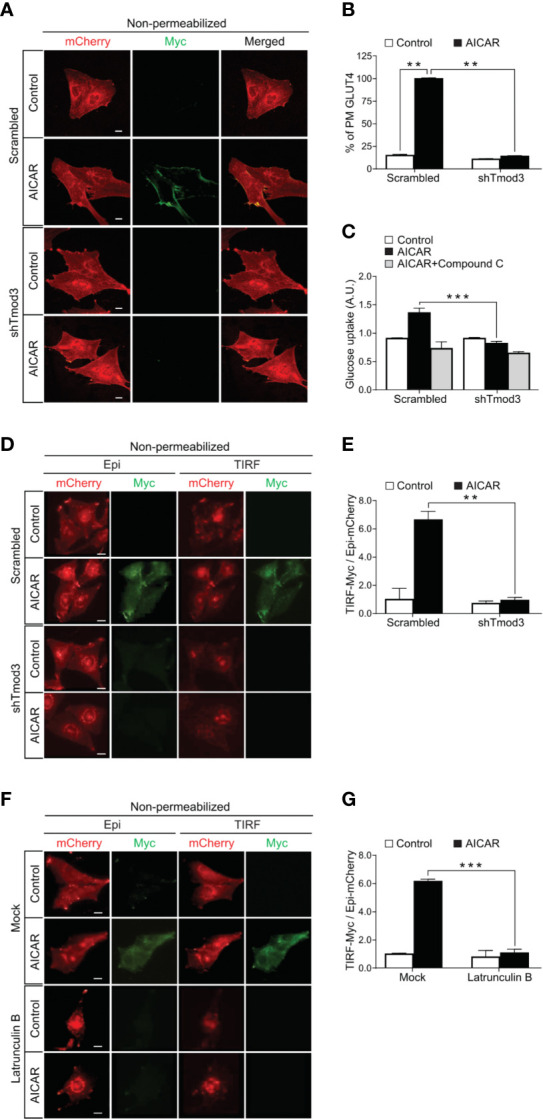
Tmod3 is essential for AMPK-mediated GLUT4 insertion into the PM and glucose uptake. **(A, B)** Diminished AICAR-stimulated GLUT4 insertion into the PM in Tmod3-KD cells. L6 myoblasts stably expressing Myc-GLUT4-mCherry were serum starved for 2 hours and treated with or without 2 mM AICAR for 30 minutes. In fixed and non-permeabilized cells, cell surface Myc-GLUT4-mcherry signals were detected with anti-Myc mouse monoclonal antibody followed by Alexa Fluor-488 goat anti-mouse secondary antibody. The samples were subjected to confocal microscopic imaging. Intensity of Myc at PM was quantified against intensity of total mCherry in the whole cell. Data in each group were normalized and expressed as a percentage of AICAR-treated scrambled control cells. Data are presented as mean ± SEM of about 100 cells in each group from three independent experiments (ANOVA with Dunnett’s multiple comparison test). **p<0.01 versus Scrambled AICAR treated group. Scale bar: 10 μm. **(C)** Impaired [^3^H]-2-Deoxyglucose uptake in Tmod3-KD L6 myoblasts. After serum starvation for 2 hours, Tmod3-KD and scrambled control cells were treated with DMSO as control, AICAR (2 mM) or AICAR plus Compound C (CC) (10 µM) for 30 minutes. Data are presented as mean ± SEM of three independent experiments (ANOVA with Dunnett’s multiple comparison test). **p<0.01 and ***p<0.001 versus AICAR treated scrambled group. **(D, E)** AICAR-induced GLUT4 translocation and insertion into the PM were examined by TIRF- and epifluorescence-microscopy in L6 myoblasts. L6 myoblasts expressing Myc-GLUT4-mCherry were serum starved for 2 hours and treated with or without AICAR (2 mM) for 30 minutes. The cells were fixed and labeled with anti-Myc antibody followed by Alexa Fluor 488-conjugated goat anti-mouse secondary antibody (green) under non-permeabilized condition. TIRF-mCherry and TIRF-Myc signals were detected using TIRF microscope. Data are presented as the ratio of cell surface TIRF-Myc signals to total Epi-mCherry and mean ± SEM of about 30 cells in each group from three sets of independent experiments (ANOVA with Dunnett’s multiple comparison test). **p<0.01 versus scrambled AICAR-treated group. Scale bar: 10 μm. **(F, G)** Latrunculin B inhibits GLUT4 insertion into the PM but not GLUT4 translocation to the periphery of the cell. L6 myoblasts expressing Myc-GLUT4-mCherry were serum starved for 2 hours and pre-treated with Latrunculin B (10 µM) for 30 minutes followed by AICAR (2 mM) for 30 minutes. The cells were fixed and labeled with anti-Myc antibody followed by Alexa Fluor 488-conjugated goat anti-mouse secondary antibody (green) under non-permeabilized condition. TIRF-mCherry and TIRF-Myc signals were detected using TIRF microscope. Data are presented as the ratio of cell surface TIRF-Myc signals to total Epi-mCherry and mean ± SEM of about 30 cells in each group from three sets of independent experiments (ANOVA with Dunnett’s multiple comparison test). **p<0.01 and ***p<0.001 versus AICAR treated Scrambled group. Scale bar: 10 μm.

Like insulin, AICAR-activated AMPK induces glucose transport (6, 26–29), which is primarily mediated by GLUT4 insertion in the PM in myocytes (30). As Tmod3 was previously shown to be involved in the regulation of insulin-stimulated GLUT4 translocation and insertion into the PM and glucose uptake in adipocytes (14), we decided to investigate whether Tmod3 was also critical in AMPK-mediated GLUT4 translocation and glucose uptake. As expected, AICAR-activated-AMPK induced apparent GLUT4 translocation and insertion into the PM (**Figures 2A, B**) and significant glucose uptake (**Figure 2C**) in scrambled control myoblasts. In contrast, Tmod3-KD myoblasts showed dramatically reduced GLUT4 surface exposure at the PM (**Figures 2A, B**) and glucose uptake (**Figure 2C**) in response to AICAR treatment. Co-treatment of AICAR with Compound C (CC), an AMPK inhibitor, abolished AICAR-induced glucose uptake in scrambled control cells (**Figure 2C**), consistent with the notion that AICAR-induced glucose uptake was mediated by AMPK. These data suggest that Tmod3 is essential for AMPK-mediated GLUT4 translocation and/or its insertion into the PM, and glucose uptake in L6 myoblasts.

### Tmod3 Is Required for GLUT4 Insertion Into the PM

To further delineate the role of Tmod3 on GLUT4 translocation and insertion into the PM, we used total internal reflection fluorescence microscopy (TIRFM) to visualize fusion events within ~200 nm from the PM, together with epifluorescence for events across the entire cell, including GLUT4 trafficking from intracellular space to the cell periphery (14). Under non-permeabilized conditions, GLUT4 inserted into the PM was quantified by the ratio of TIRF-Myc fluorescence to Epi-mCherry fluorescence (14). In the control cells, AICAR stimulation led to both significant GLUT4 translocation to the TIRF zone near the PM and insertion into the PM, and surface exposure of the Myc epitope as evidenced by the red and green fluorescence signals (**Figures 2D, E**). In contrast, Tmod3-KD myoblasts displayed diminished GLUT4 insertion into the PM, although GLUT4 trafficking to the TIRF zone was largely unaffected (**Figures 2D, E**). These results suggest that KD of Tmod3 affects GLUT4 vesicle fusion with the PM but not its trafficking from the intracellular space to the cortical area of cell periphery in L6 myoblasts.

To corroborate the above findings, we applied Latrunculin B treatment to distinguish the effect of Tmod3 KD on GLUT4 translocation and insertion into the PM, as Latrunculin B has been shown to disrupt GLUT4 vesicle fusion with the PM but not its intracellular translocation (14). Incubation of the cells with Latrunculin B causes dispersion of the actin filaments and much of cortical actin (31). Interestingly, under Latrunculin B treatment, GLUT4 appeared to be mostly concentrated perinuclear and partially dispersed. As shown in the representative images (**Figure 2F**) and quantifications (**Figure 2G**), although the disruption of cortical F-actin with Latrunculin B resulted in accumulation of GLUT4 signal in the TIRF zone, concurrently significant reduction of GLUT4 surface exposure at the PM was observed despite of AMPK activation. Together, these results indicate that Tmod3 is required for GLUT4 insertion into the PM but not trafficking to the cell periphery.

### Tmod3 Is Phosphorylated by AMPK at Ser25

**Figure 3:**
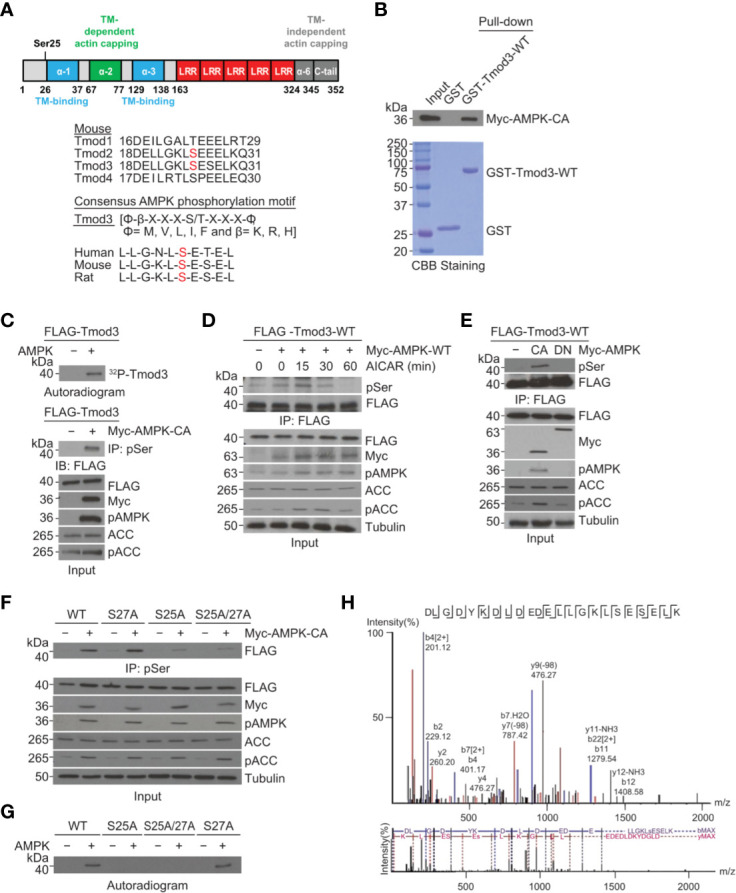
Tmod3 is phosphorylated by AMPK at Ser25. **(A)** Schematic diagram of different domains of Tmod3 with identified phosphorylation site as shown in the upper panel. AMPK consensus sequence is present in both Tmod2 and Tmod3 but not in Tmod1 or Tmod4 as shown in the lower panel. **(B)** GST pull-down assay showing AMPK-Tmod3 interaction. GST-fused Tmod3-WT was purified from BL21 bacterial cells and incubated with lysates of HEK293T cells expressing Myc-AMPK-WT. The interaction was detected using anti-Myc antibody. **(C)** *In vitro* kinase assay showing Tmod3 phosphorylation by AMPK. Purified full-length mouse Tmod3 was incubated with recombinant AMPK and γ-^32^P-ATP. The samples were analyzed by SDS-PAGE and autoradiography. **(D)** Kinetics of AICAR-induced phosphorylation of Tmod3. AICAR induced phosphorylation of Tmod3 in AMPK-dependent manner. HEK293T cells co-expressing FLAG-Tmod3-WT and Myc-AMPK-WT were either untreated or treated with AICAR (2 mM) for indicated periods of time. Anti-FLAG M2 affinity gel was used for immuno-precipitation of phosphorylated Tmod3 from the cell lysates followed by immuno-blotting with anti-Phospho Serine antibody. **(E)** Tmod3 interacts with and is phosphorylated by constitutively active (CA) AMPK-α2 but not kinase-inactive (DN) AMPK. AMPK-mediated phosphorylation of Tmod3 in HEK293T cells following co-expression of FLAG-Tmod3-WT with AMPK-CA or AMPK-DN were detected by pull-down using anti-FLAG M2 affinity gel followed by immuno-blotting with anti-Phospho Serine antibody. **(F)** AMPK phosphorylates Tmod3 at Ser25. FLAG-Tmod3 and its mutants co-expressed with Myc-AMPK-CA were pulled down using anti-phospho-Serine antibody conjugated to protein A/G Sepharose beads and phosphorylated Tmod3 signals were detected using anti-FLAG antibody. **(G)** Ser25 is the phosphorylation target of AMPK as demonstrated by autoradiography assay. **(H)** Identification of Ser25 residue in Tmod3 as the phosphorylation site of AMPK by mass spectrometry. Data are representative of three independent experiments except for panel **(H)**.

To investigate the functional role of Tmod3 in AMPK-mediated GLUT4 insertion into the PM and glucose uptake, we decided to first determine whether Tmod3 was an AMPK substrate, and if so, to identify the phosphorylation site. We performed sequence analysis and discovered the presence of an AMPK consensus phosphorylation motif [LLGKLS*ESEL, Ser25] near the α-helix1 region at the N-terminus of Tmod3 (**Figure 3A**). The phosphorylation motif is present in Tmod2 and Tmod3, but not in Tmod1 or Tmod4 (**Figure 3A**). Sequence analysis of Tmod3 shows that the consensus AMPK phosphorylation motif is conserved in human, mouse and rat Tmod3 sequence (**Figure 3A**). We then examined and confirmed a direct interaction between Tmod3 and AMPK by standard GST-pull-down assay (**Figure 3B**). To determine whether Tmod3 was a direct AMPK substrate, we performed an *in vitro* kinase assay and found that Tmod3-WT was phosphorylated in the presence of AMPK (**Figure 3C**). To ascertain Tmod3 as a potential AMPK substrate, we co-expressed FLAG-Tmod3-WT with or without mouse Myc-AMPK-α2-WT in HEK293T cells and treated the cells with AICAR. As shown in **Figure 3D**, Tmod3 phosphorylation rapidly peaked at 15 minutes under AICAR treatment. As expected, AMPK phosphorylation was readily detected throughout AICAR treatment (**Figure 3D**). Similarly, FLAG-Tmod3-WT was co-expressed with either constitutively active (Myc-AMPK-α2-CA) or kinase inactive (Myc-AMPK-α2-DN, K45R mutation) AMPK and subjected to immuno-precipitation using anti-FLAG M2 affinity gel. FLAG-Tmod3-WT was phosphorylated in the presence of constitutively active form of AMPK but not the kinase-inactive form of AMPK (**Figure 3E**). Finally, *in vivo* studies in C57BL/6 mice (**Supplementary Figure 4A**) and in L6 myotubes (**Supplementary Figure 4B**) showed increased Tmod3 phosphorylation under AMPK activation. These data collectively demonstrate that Tmod3 is a novel substrate for AMPK signaling.

To confirm whether the identified AMPK phosphorylation motif is the target site for AMPK in Tmod3, we expressed FLAG-Tmod3-WT and its mutants in HEK293T cells in the presence or absence of Myc-AMPK-α2-CA, pulled down using anti-phospho-serine antibody conjugated to protein A/G Sepharose beads, and immunoblotted to detect phosphorylated Tmod3 using anti-FLAG antibody. As shown in **Figure 3F**, Tmod3-WT and Tmod3-S27A, but not Tmod3-S25A or Tmod3-S25A/S27A, were phosphorylated, confirming that Ser25 is the site of AMPK phosphorylation on Tmod3. This finding was further corroborated by autoradiography (**Figure 3G**) and mass spectrometry analysis (**Figure 3H**). Taken together, these results experimentally demonstrate that Tmod3 is directly phosphorylated by AMPK at Ser25.

### Tmod3 Phosphorylation Is Required for AMPK-Mediated GLUT4 Insertion Into the PM and Glucose Uptake

**Figure 4:**
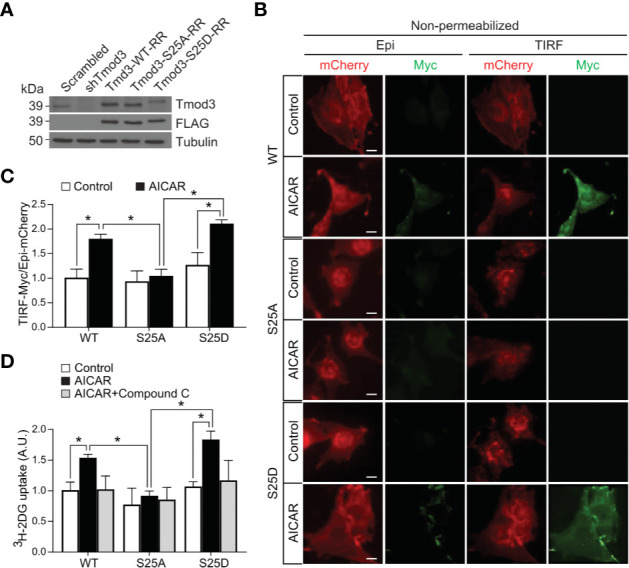
Tmod3 phosphorylation is required for AMPK-mediated GLUT4 insertion into the PM and glucose uptake. **(A)** Western blot analysis showing endogenous Tmod3 expression in scrambled control L6 myoblasts and FLAG-tagged Tmod3 mutants in Tmod3-KD L6 myoblasts. Tmod3 expressions were detected by anti-Tmod3 rabbit antibody and anti-FLAG mouse antibody. Tubulin was used as the loading control. **(B, C)** Tmod3-WT and phospho-mimetic mutant Tmod3-S25D promotes GLUT4 insertion into the PM upon AMPK activation. L6 myoblasts co-expressing Myc-GLUT4-mCherry and Tmod3-WT or mutants were serum starved for 2 hours and treated with or without AICAR (2 mM) for 30 minutes. The cells were fixed and labeled with anti-Myc antibody followed by Alexa Fluor 488-conjugated goat anti-mouse secondary antibody (green) under non-permeabilized condition. TIRF-mCherry and TIRF-Myc signals were detected using TIRF microscope. Data are presented as the ratio of cell surface TIRF-Myc signals to total Epi-mCherry and mean ± SEM of 100 cells in each group from three sets of independent experiments (ANOVA with Dunnett’s multiple comparison test). ^*^p<0.05. Scale bar: 10 μm. **(D)** L6 myoblasts expressing Tmod3-WT or mutants were serum starved for 2 hours, treated with DMSO as control, AICAR (2 mM) and Compound C (CC) (10 µM) as inhibitor for 30 minutes for [^3^H]-2-Deoxyglucose uptake assay. Data are presented as mean ± SEM of three independent experiments (ANOVA with Dunnett’s multiple comparison test). ^*^p<0.05.

To determine the effects of Tmod3 phosphorylation at Ser25 on AMPK-mediated GLUT4 insertion into the PM and glucose uptake, we stably expressed WT or mutants in Tmod3-depleted L6 myoblasts (**Figure 4A**) using lenti-viral particles and performed TIRF-based Myc-GLUT4-mCherry assay under non-permeabilized conditions. Upon activation of AMPK, significant GLUT4 insertion into the PM was detected in Tmod3-depleted myoblasts expressing Tmod3-WT or phospho-mimetic mutant (S25D) as evidenced by strong green signals from antibody recognition of surface-exposed Myc epitope (**Figures 4B, C**). In contrast, Tmod3-KD myoblasts expressing phospho-defective mutant (S25A) showed nearly undetectable GLUT4 surface exposure (**Figures 4B, C**), suggesting that Tmod3 phosphorylation is required for GLUT4 insertion into the PM in response to AMPK activation. Consequently, significantly enhanced glucose transport capacity was observed in Tmod3-S25D expressing myoblasts upon AMPK activation, whereas almost no AMPK-enhanced glucose uptake was detected in Tmod3-S25A expressing myoblasts (**Figure 4D**). As expected, Compound C treatment led to significant reduction in glucose uptake in cells expressing Tmod3-WT or its mutants (**Figure 4D**). These data show that Tmod3 phosphorylation is essential in AMPK-mediated GLUT4 insertion into the PM and glucose uptake in L6 myoblasts.

### AMPK-Mediated Tmod3 Phosphorylation Regulates Cortical F-Actin Remodeling

**Figure 5:**
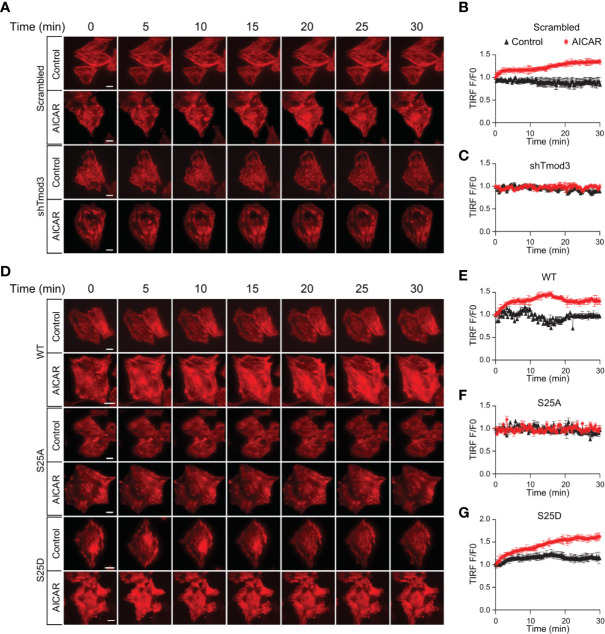
AMPK-mediated Tmod3 phosphorylation regulates cortical F-actin remodeling. **(A)** Representative time-lapse images of F-actin remodeling in Tmod3-KD or scrambled control L6 myoblasts stably expressing Lifeact-tdTomato. The cells were serum starved, treated with or without AICAR (2 mM) and imaged for 30 minutes at an interval of 15 seconds. Scale bar: 10 μm. **(B, C)** Representative examples of TIRF-Lifeact-tdTomato F-actin remodeling analysis. Measurement on the ventral regions away from the edge after the removal of background fluorescence, TIRF intensities of ROIs measured over time were normalized to the intensity measured at the zero time point, averaged and plotted against the time to indicate the time course of F-actin remodeling. N = 8-10 cells per condition were analyzed. **(D)** Representative time-lapse images of F-actin remodeling in L6 myoblasts stably expressing Lifeact-tdTomato and Tmod3-WT or mutants under AICAR-stimulated condition. The cells were serum starved, treated with or without AICAR (2 mM) and imaged for 30 minutes at an interval of 15 seconds. Scale bar: 10 μm. **(E–G)** Representative examples of TIRF-Lifeact-tdTomato F-actin remodeling analysis. Measurement on the ventral regions away from the edge after the removal of background fluorescence, TIRF intensities of ROIs measured over time were normalized to the intensity measured at zero time point, averaged and plotted against the time to indicate the time course of F-actin remodeling. N = 8-10 cells per condition were analyzed.

We previously demonstrated that phosphorylation of Tmod3, albeit at a different site (Ser71), regulates insulin-induced F-actin remodeling (14). To examine whether AMPK-stimulated phosphorylation of Tmod3 at Ser25 regulates GLUT4 translocation by inducing F-actin remodeling, we first established L6 myoblasts stably expressing Lifeact-tdTomato (**Supplementary Figure 5A**). Lifeact, a 17 amino-acid peptide, when fused to tdTomato, stains F-actin structures without interfering actin dynamics *in vitro* or *in vivo* (32). Upon AMPK activation, increased F-actin remodeling was observed in WT cells as shown by increased Lifeact-tdTomato fluorescence under TIRFM (**Figures 5A, B** and **Supplementary Figure 5B** and **Supplementary Movies 1A, B**). In contrast, F-actin remodeling was largely absent in response to AMPK activation in Tmod3-KD cells (**Figures 5A, C** and **Supplementary Figure 5C** and **Supplementary Movies 1C, D**), suggesting that Tmod3 is required for AMPK-induced F-actin remodeling in L6 myoblasts. We next generated L6 myoblasts stably co-expressing Lifeact-tdTomato and Tmod3-WT or Tmod3 phosphorylation mutants. Compared to mock treatment (control), AMPK activation by AICAR induced cortical F-actin remodeling as shown by increased Lifeact-tdTomato fluorescence under a TIRF microscope in cells expressing Tmod3-WT or Tmod3-S25D, but not in cells expressing Tmod3-S25A (**Figures 5D–G** and **Supplementary Figures 5D–F** and **Supplementary Movies 2A–F**). Together, these data show that AMPK-induced phosphorylation of Tmod3 at Ser25 induces cortical F-actin remodeling.

### Phosphorylation of Tmod3 Does Not Regulate Core Tmod3: Tropomyosin: Actin Complex Formation

**Figure 6:**
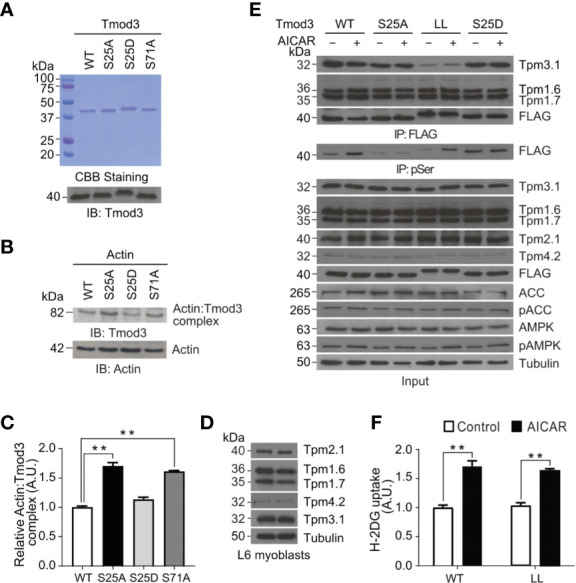
AMPK-mediated Tmod3 phosphorylation regulates its binding to monomeric G-actin independent of Tmod3: Tropomyosin interaction. **(A)** Recombinant proteins of Tmod3-WT and mutants were prepared and visualized by Coomassie Brilliant Blue staining and confirmed by Western blotting using anti-Tmod3 rabbit antibody. **(B)** Recombinant proteins of Tmod3-WT and mutants were subjected to *in vitro* actin cross-linking assay in the presence or absence of G-actin at equimolar ratio and cross-linked with EDC/sulfo-NHS cross-linkers for 45 minutes at room temperature. The cross-linking assays were terminated by adding 2X SDS sample buffer, followed by Western blotting with anti-β-actin rabbit antibody and anti-Tmod3 rabbit antibody. Data are representative of three independent experiments. **(C)** Quantification of actin: Tmod3 complex by densitometry. Data are presented as mean ± SEM of three independent experiments (ANOVA with Dunnett’s multiple comparison test). ^**^p<0.01. **(D)** Expression of different tropomyosin isoforms in L6 myoblasts. **(E)** L6 myoblasts stably expressing FLAG-Tmod3-WT and mutants were serum starved for 2 hours and treated with or without 2 mM AICAR for 30 minutes. Anti-FLAG M2 affinity gel was used for immuno-precipitation followed by immuno-blotting with mouse monoclonal anti-α-Tm9d, CG1 and γTm9d, rabbit polyclonal δTm9d and mouse anti-FLAG antibodies. Data are representative of three independent experiments. **(F)** L6 myoblasts expressing Tmod3-WT and Tmod3-LL were serum starved for 2 hours, treated with DMSO as control and AICAR (2 mM) for 30 minutes for [^3^H]-2-Deoxyglucose uptake assay. Data are presented as mean ± SEM of three independent experiments (ANOVA with Dunnett’s multiple comparison test). ^**^p<0.01.

A characteristic feature of F-actin capping proteins is their ability to bind with monomeric G-actin (33). We thus examined the ability of Tmod3 to bind with G-actin by using an *in vitro* actin-cross-linking assay, followed by SDS-PAGE and immuno-blotting with anti-Tmod3 and anti-actin antibodies (**Figures 6A, B**). Compared with Tmod3-WT, the phospho-defective mutant Tmod3-S25A showed increased Tmod3: Actin complex formation (**Figure 6C**), to a similar extent as Tmod3-S71A (14). Interestingly, the phosphomimetic mutant Tmod3-S25D had no effect on Actin: Tmod3 complex formation. The finding that phosphorylation of Tmod3 at Ser25 has little effect on its binding to G-actin suggests that the observed cortical F-actin remodeling mediated by Tmod3 Ser25 phosphorylation is not due to altered Tmod3:Actin complex formation.

Within the N-terminal domain of Tmod3, α-helix-1 and α-helix-3 were proposed to be involved in Tmod3 interaction with tropomyosin, while α-helix-2 in Tmod3 binding with G-actin and tropomyosin-dependent F-actin capping (34–36). The binding affinity of tropomodulin to actin filaments increases in the presence of a tropomyosin (37). We previously demonstrated that Tmod3 could interact with Tpm3.1, the shorter form of γTm (tropomyosin), but not with other isoforms of tropomyosin in adipocytes under insulin-stimulated conditions (14). However, Tmod3 mutant with defective tropomyosin interacting domains, Tmod3-L29G/L134D (Tmod3-LL), completely lost binding with Tpm3.1 indicating the importance of Tmod3:Tpm3.1 interaction for efficient insulin-stimulated GLUT4 translocation and glucose uptake in adipocytes (14). Consistent with previous studies, we found that L6 myoblasts expressed at least the following tropomyosin isoforms: Tpm2.1, Tpm1.6, Tpm1.7, Tpm4.2 and Tpm3.1 (**Figure 6D**) (38). To identify the cognate interacting partner for Tmod3, we generated stable L6 myoblasts expressing FLAG-Tmod3-WT or Tmod3 mutants and performed co-immunoprecipitation analysis by using anti-FLAG M2 affinity gel, followed by antibody detection of different tropomyosin isoforms. We found that Tmod3 exhibited apparent interaction with Tmp1.6, Tpm1.7 and Tpm3.1 but not with Tpm2.1 or Tpm4.2 (**Figure 6E**). Consistent with our previous report (14), Tmod3-L29G/L134D (Tmod3-LL) mutant showed significantly decreased binding to Tpm3.1 under both mock and AMPK activated conditions. Interestingly, the Tmod3: tropomyosin interaction was not affected by the AMPK-induced Tmod3 phosphorylation at Ser25, which is at the start of α-helix-1 (**Figure 6E**). Expression of Tmod3-LL mutant inhibits insulin-stimulated glucose uptake in adipocytes (14), but in case of L6 myoblasts expressing Tmod3-LL significant increase in glucose uptake was observed under AMPK activation (**Figure 6F**). Collectively, these data show that AMPK-induced phosphorylation of Tmod3 promotes GLUT4 insertion into the PM and glucose uptake in L6 myoblasts results from a mechanism independent of core Tmod3: tropomyosin: actin complex formation.

## Discussion

Both contraction and insulin acutely stimulate recruitment of existing GLUT4 to the PM of the skeletal muscle and adipocytes independent of transcription and translation (39, 40). Recent discovery of Tmod3 as a novel Akt2 substrate has provided a molecular mechanism underlying insulin-stimulated GLUT4 insertion into the PM and glucose uptake in adipocytes (14). In the muscle, glucose transport occurs *via* two major pathways, insulin-dependent and insulin-independent signaling pathways. In the insulin-independent pathway, contraction of skeletal muscles activates AMPK, which stimulates glucose uptake by promoting GLUT4 translocation and insertion into the PM (1, 41). Contraction and direct activation of AMPK-mediated mechanisms remain intact in insulin-resistant individuals (42–44). As such, the molecules at which AMPK- and insulin-signaling pathways converge in the stimulation of GLUT4 insertion into the PM may provide drug targets to bypass the failure in insulin-mediated GLUT4 translocation and insertion into the PM, such as in insulin-resistant patients and animals.

Insulin signaling and its regulation of GLUT4 trafficking and glucose uptake, including identification of insulin-Akt substrates and understanding of their mechanism of action, have been under intense investigations over the last three decades. In the case of GLUT4 regulation, several key target substrates of Akt have been identified and characterized, for example AS160, Synip, myosin Va, PIKfyve, CDP138, Grp1 and Tmod3 (14, 21, 45–49). These studies have provided significant insights in understanding how insulin signaling regulates GLUT4 translocation and insertion into the PM. However, much less is known regarding mechanisms involved in GLUT4 translocation and glucose transport other than the insulin signaling pathway.

In this report, we identify that Tmod3 is a novel AMPK substrate and provide evidence to show that phosphorylation of Tmod3 is required for AMPK-mediated GLUT4 insertion into the PM and glucose uptake. First, upon silencing of Tmod3 in L6 myoblasts, AMPK-mediated GLUT4 insertion into the PM and glucose uptake were largely abolished. Second, sequence analysis, *in vitro* and *in vivo* biochemical assays, together with mass spectrometry analysis identified Ser25 as the AMPK phosphorylation target site on Tmod3. And third, in Tmod3-KD cells, stable expression of Tmod3-WT or Tmod3-S25D, but not Tmod3-S25A, was able to rescue the defect in AMPK-mediated GLUT4 insertion into the PM and glucose uptake. Tmod3 has been shown to be phosphorylated by Akt2 at Ser71 under insulin stimulation, however, mutation in Ser25 to Ala showed no effect on the Tmod3 phosphorylation Akt2 activation suggesting that Tmod3 phosphorylation at these two different sites are regulated by two different pathways (14). Although AS160 was previously proposed to be a substrate for AMPK and AS160 phosphorylation by AMPK was shown to be involved in the regulation of glucose uptake, AMPK-phosphorylation of AS160 very likely acts during GLUT4 translocation from intracellular space to the periphery, at steps upstream of GLUT4 insertion into the PM, similar to its role under insulin stimulation (9). This notion is supported by the fact that AS160 phosphorylation by AMPK could only partially bypass insulin resistance, which suggests that a more distal target of AMPK activation plays a more direct role in AMPK-mediated GLUT4 insertion into the PM and glucose uptake (27, 50). Considering that Tmod3 phosphorylation by AMPK acts at or near membrane fusion steps, we believe that Tmod3 fits the description and is the first AMPK substrate that is directly involved in the regulation of GLUT4 insertion into the PM.

Exercise enhances insulin-stimulated GLUT4 translocation and glucose uptake (51). The underlying mechanisms responsible for exercise-induced improvements in insulin sensitivity are still unclear. However, during exercise contraction-mediated improvements in insulin sensitivity are associated with increase in AMPK activity, which promotes GLUT4 translocation to the plasma membrane (42) and increases glucose uptake (27). Similarly, in post exercise state, increase in Akt deactivates TCB1D4 and increases GLUT4 translocation to the plasma membrane (52). Here we report Tmod3 as a novel AMPK substrate and an essential mediator of AMPK-dependent GLUT4 translocation and glucose uptake in myoblasts. In our previous study we have reported that phosphorylation of Tmod3 by Akt2 mediates GLUT4 translocation and glucose uptake in adipocytes under insulin stimulation (14). Given that Tmod3 has both AMPK- and Akt2-mediated phosphorylation sites, its phosphorylation may be involved in such insulin sensitivity enhancement upon exercise.

The actin cytoskeleton remodeling is important for insulin-induced GLUT4 translocation in L6 myotubes (53–55) and epitrochlearis skeletal muscle of rat (56). The small Rho family GTPase Rac1 has been shown to play an important role in this aspect (53–55, 57–59). Activation of Rac1 has been shown to be independent of activating AMP resulted by increased metabolic stress (60, 61) whereas AMPK is activated by this increase in intracellular AMP level (62). However, Rac1 can be activated rapidly by mechanical stress or stretching during muscle contraction (63), a stimulus that does not activate AMPK (64, 65). Similarly, Rac1 and AMPK mediate glucose uptake in muscle but stretch-induced glucose transport is mediated by Rac1 only (63), not by AMPK (65, 66). It has been shown that AMPK and Rac1 together contribute for muscle glucose transport during *ex vivo* contraction, whereas only Rac1 regulates muscle glucose uptake during submaximal exercise *in vivo* (67). Rac1 and AMPK thus rely on distinct signals for activation during muscle contraction indicating that AMPK and Rac1 are activated *via* different and independent mechanisms in muscle. It has been shown that exercise in mice and humans activates Rac1 in muscles and chemical inhibition or silencing of Rac1 in muscle leads to partial impairment in contraction-induced glucose uptake in mouse muscle, indicating the role of cytoskeletal components in contraction-induced glucose uptake in muscle (51, 68). As such, Tmod3, which regulates actin filaments by capping filaments at their pointed ends (34), may regulate AMPK-mediated GLUT4 translocation through F-actin remodeling. Consistent with this notion, we observed significant cortical F-actin enrichment and increased rate of both ventral and peripheral F-actin remodeling in response to AMPK activation (**Figures 5A, B**, and **Supplementary Figure 5B**). In contrast, change of F-actin remodeling rate was not detected in Tmod3-KD myoblasts under the same condition (**Figures 5A, C**, and **Supplementary Figure 5C**). Moreover, the cellular response of cortical F-actin enrichment and F-actin remodeling rate in response to AMPK activation was restored by exogenous expression of Tmod3-WT or Tmod3-S25D (**Figures 5D–G**, and **Supplementary Figures 5D, F**), but not by Tmod3-S25A, closely correlating to their ability to rescue the defect of GLUT4 insertion into the PM and glucose uptake due to Tmod3 KD (**Figures 4B–D**).

**Figure 7:**
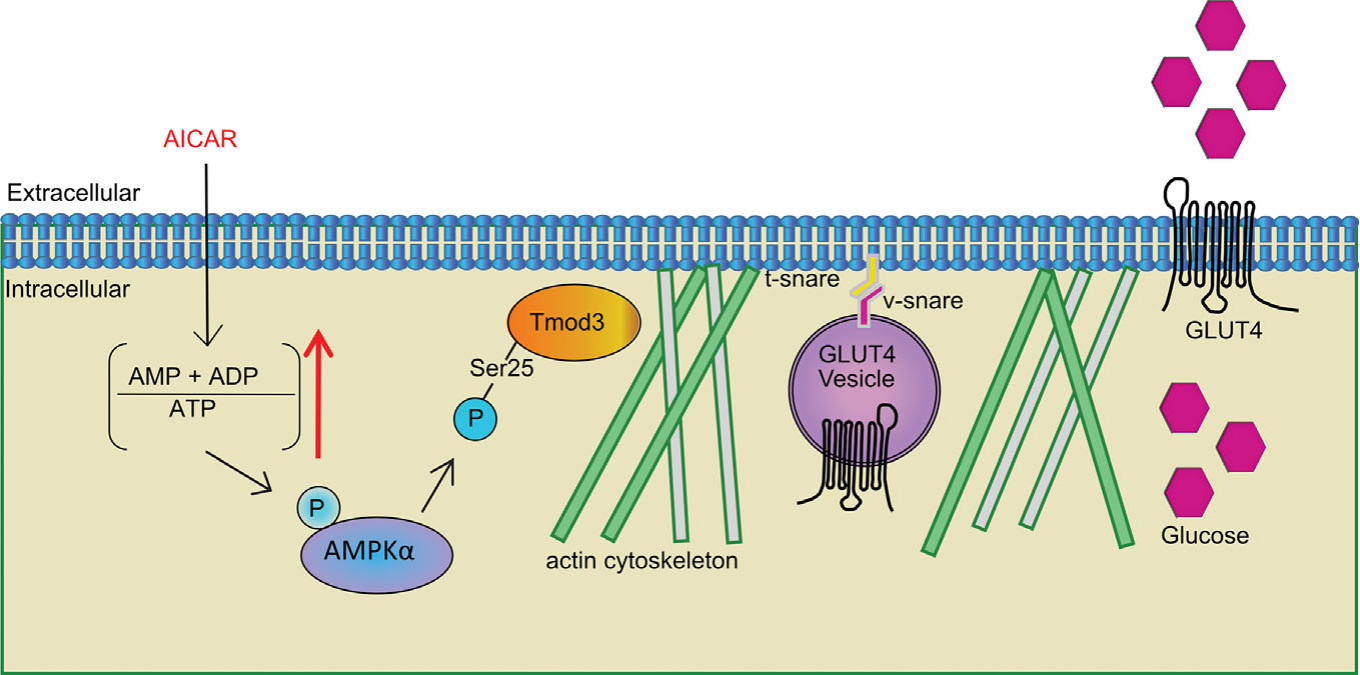
Tmod3 as an AMPK substrate bridging AMPK activation and GLUT4 insertion into the PM and glucose uptake in myoblasts. Phosphorylation of Tmod3 by AMPK promotes cortical F-actin enrichment and remodeling, thereby facilitating AMPK-mediated GLUT4 insertion into the PM and glucose uptake in myoblasts. The stimuli other than AICAR, may be expected to phosphorylate Tmod3, since they activate AMPK, but this will require further experimentation.

Structurally, Tmod3 contains an N-terminal domain with three functional α-helices and caps the pointed ends of actin filaments (69–71). α-helix-1 and α-helix-3 bind to tropomyosin (69, 70) and α-helix-2 caps pointed ends of actin filament in a tropomyosin-dependent manner (70, 72) forming a co-polymer along the length of actin filament (73). There are about 40 tropomyosins, and tropomodulins bind to specific types of tropomyosin (74). The type of tropomodulin-tropomyosin pair regulates its interactions with actin binding proteins and myosin motors, thereby determines the functional capacity of the filament (74–76). Since both the α-helix-1 and α-helix-3 of Tmod3 are necessary for the interaction with a tropomyosin, Tmod3 mutant harboring both L29G in the α-helix-1 and L134D in the α-helix-3 (Tmod3-LL), equivalent to L27E and I131D mutations in Tmod1 (77), has completely lost its binding to Tpm3.1 in adipocytes (14). Consistently, Tmod3-LL exhibited diminished binding to Tpm3.1 in myoblasts under both basal and AMPK activation conditions (**Figure 6E**). More importantly, Tmod3-WT and its phosphorylation mutants (S25A and S25D) also did not show altered interaction with tropomyosins under AMPK activation (**Figure 6E**). In addition to this, expression of Tmod3-LL significantly increased glucose uptake under AMPK activation (**Figure 6F**) showing that Tmod3-LL does not affect AMPK-stimulated glucose uptake, in contrast to decreased glucose uptake under insulin stimulation in adipocytes (14). The finding that Tmod3 interaction with Tpm3.1 is not dependent on AMPK-induced phosphorylation of Tmod3 suggests that the Tmod3: tropomyosin interaction is not a limiting step in AMPK-mediated GLUT4 insertion into the PM and glucose uptake. Based on our findings and the above discussion, a plausible model emerges that Tmod3 phosphorylation at Ser25 regulates cortical F-actin enrichment and remodeling to promote GLUT4 insertion into the PM and glucose uptake in response to AMPK activation through a mechanism independent from the regulation of core Tmod3:tropomyosin:actin complex (**Figure 7**). This may involve additional actin regulatory proteins, such as cofilin and gelsolin, but the detailed mechanism remains to be determined.

## Data Availability Statement

The raw data supporting the conclusions of this article will be made available by the authors, without undue reservation.

## Ethics Statement

The animal study was reviewed and approved by Institutional Animal Care and Use Committee (IACUC #161122).

## Author Contributions

MS, C-YL and WH conceived the project and designed the experiments. MS and RR designed *in-vitro* experiments. MS performed molecular experiments, bioinformatics analysis, imaging and *in-vitro* and *in-vivo* experiments. XB performed Mass Spectrometry and data analysis. MS, RR and WH wrote the manuscript. All authors participated in the discussions of the manuscript. All authors contributed to the article and approved the submitted version.

## Funding

The current research study was supported by A*STAR (Agency for Science, Technology and Research) Biomedical Research Council. 

## Conflict of Interest

The authors declare that the research was conducted in the absence of any commercial or financial relationships that could be construed as a potential conflict of interest.

## Abbreviations

AICAR, 5-Aminoimidazole-4-carboxamide ribonucleotide; AMP, Adenosine monophosphate; AMPK, 5’ adenosine monophosphate-activated protein kinase; ATCC, American Type Culture Collection; ATP, Adenosine Triphosphate; CA, Constitutively Active; DMEM, Dulbecco’s Modified Eagle Media; DN, Dominant Negative; GLUT4, Glucose Transporter Type 4; GST, Glutathione S-transferase; IACUC, Institutional Animal Care and Use Committee; KD, Knock Down; MS, Mass Spectrometry; PAGE, Polyacrylamide Gel Electrophoresis; PBS, Phosphate Buffer Saline; PI3K, Phosphoinositide 3-Kinase; PM, Plasma membrane; SDS, Sodium Dodecyl Sulphate; TIRFM, Total Internal Reflection Fluorescence Microscopy; Tmod3, Tropomodulin 3; WT, Wild Type.
