# Supplementary figures and images for: Tmod3 Phosphorylation Mediates AMPK-Dependent GLUT4 Plasma Membrane Insertion in Myoblasts
Source: Front Endocrinol (Lausanne). 2021 Apr 20;12:653557. doi: 10.3389/fendo.2021.653557 (PMC8095187; doi:10.3389/fendo.2021.653557)

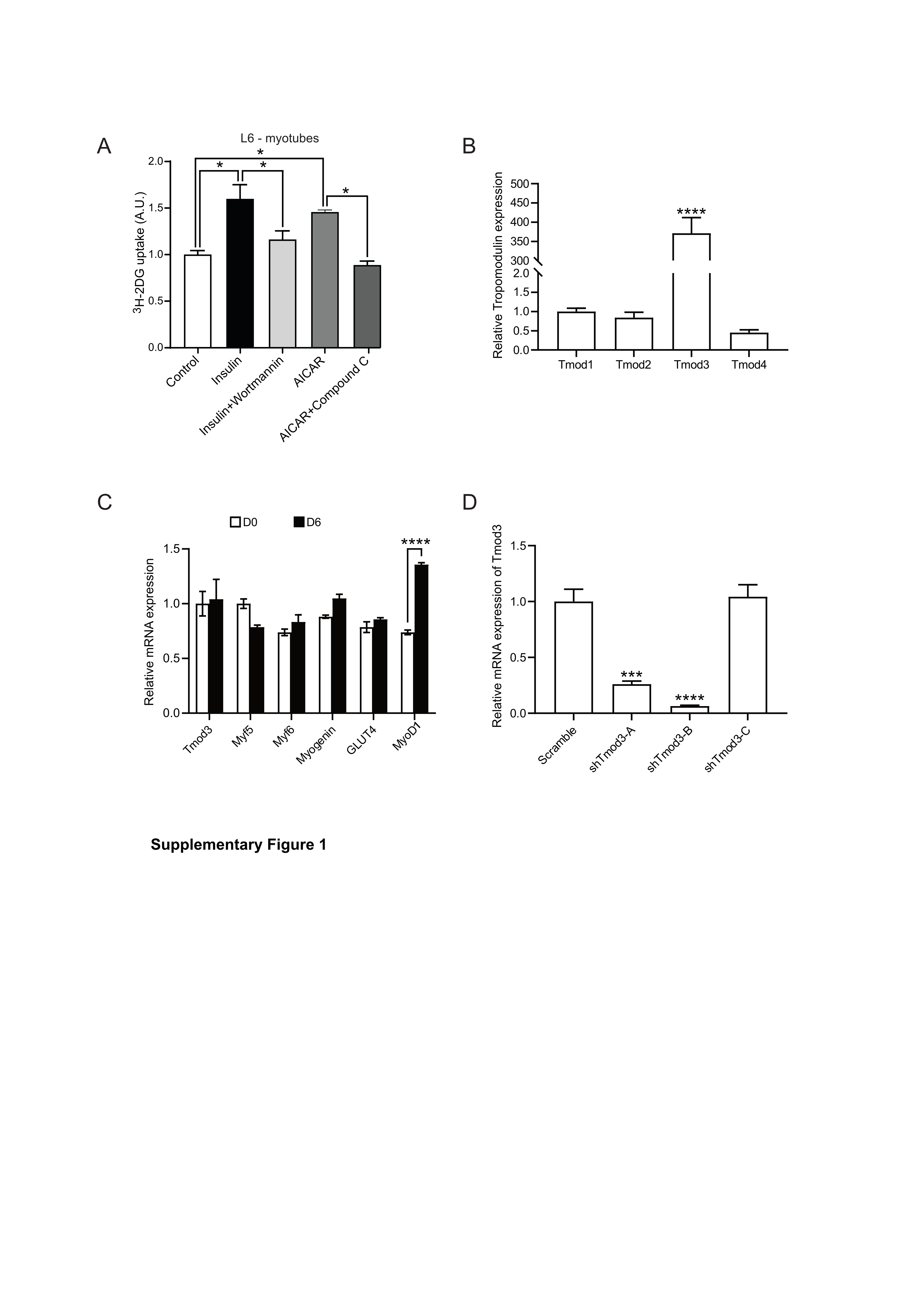

Supplement: Supplementary file 1 [file Image_1.tiff]

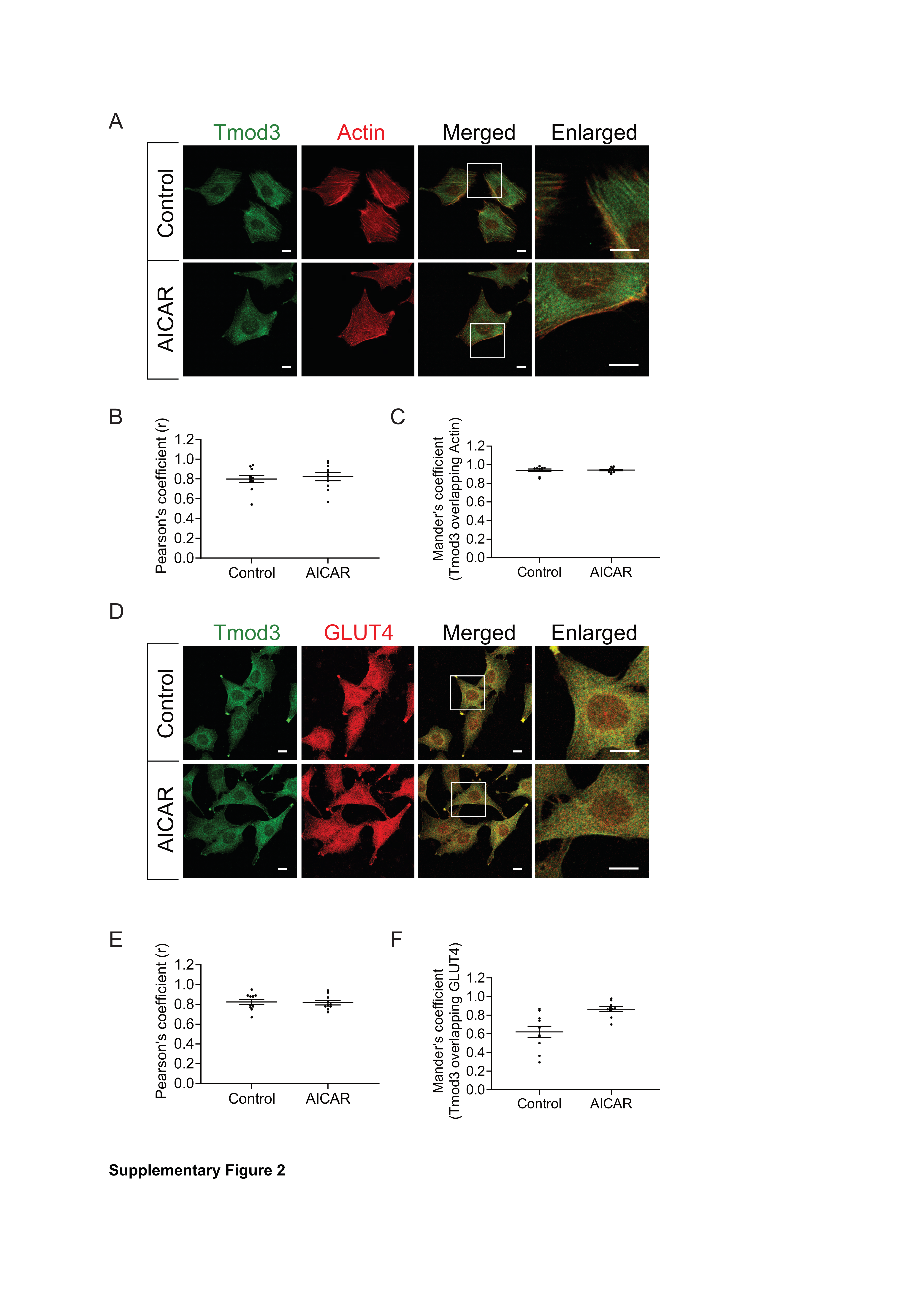

Supplement: Supplementary file 2 [file Image_2.tiff]

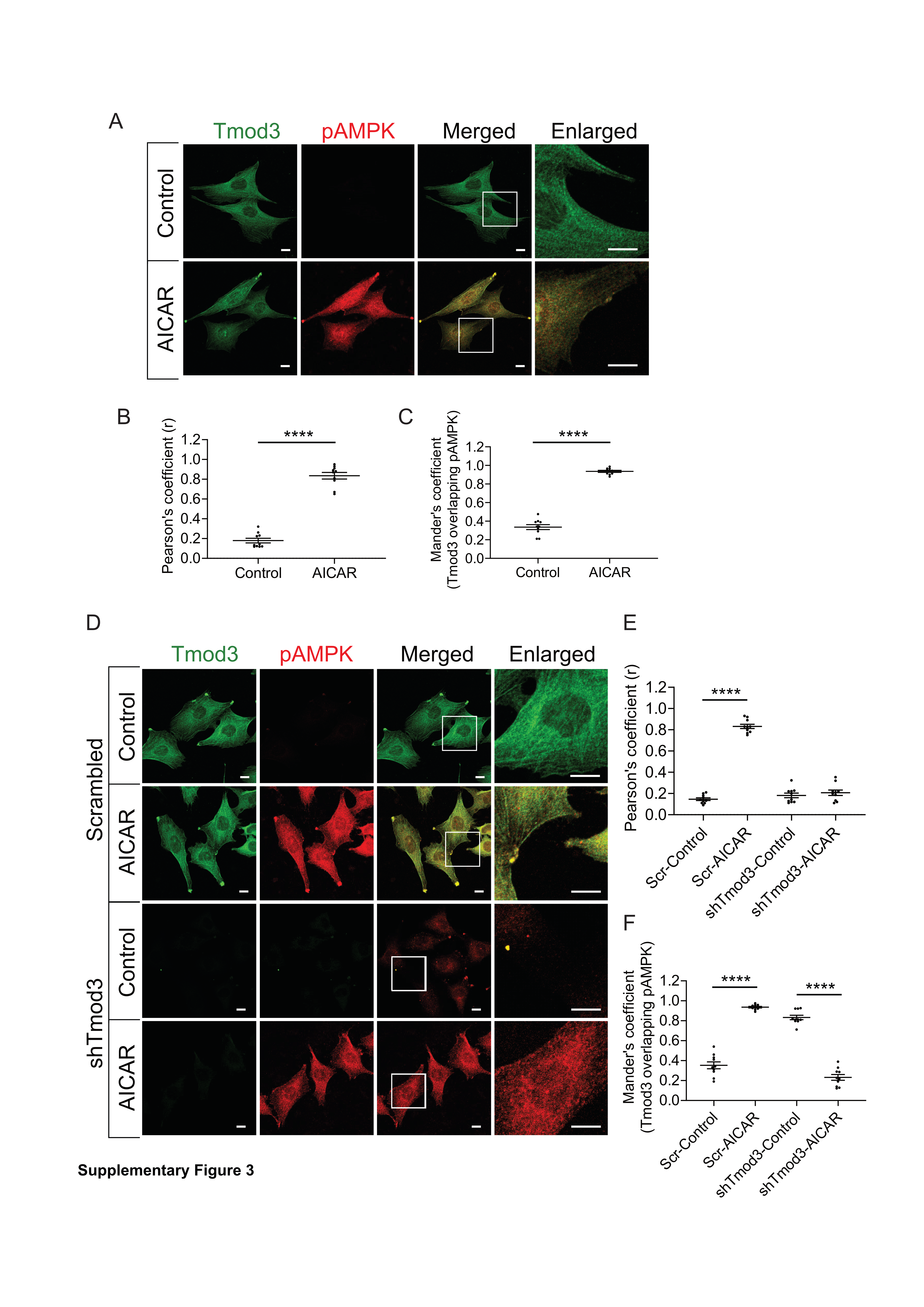

Supplement: Supplementary file 3 [file Image_3.tiff]

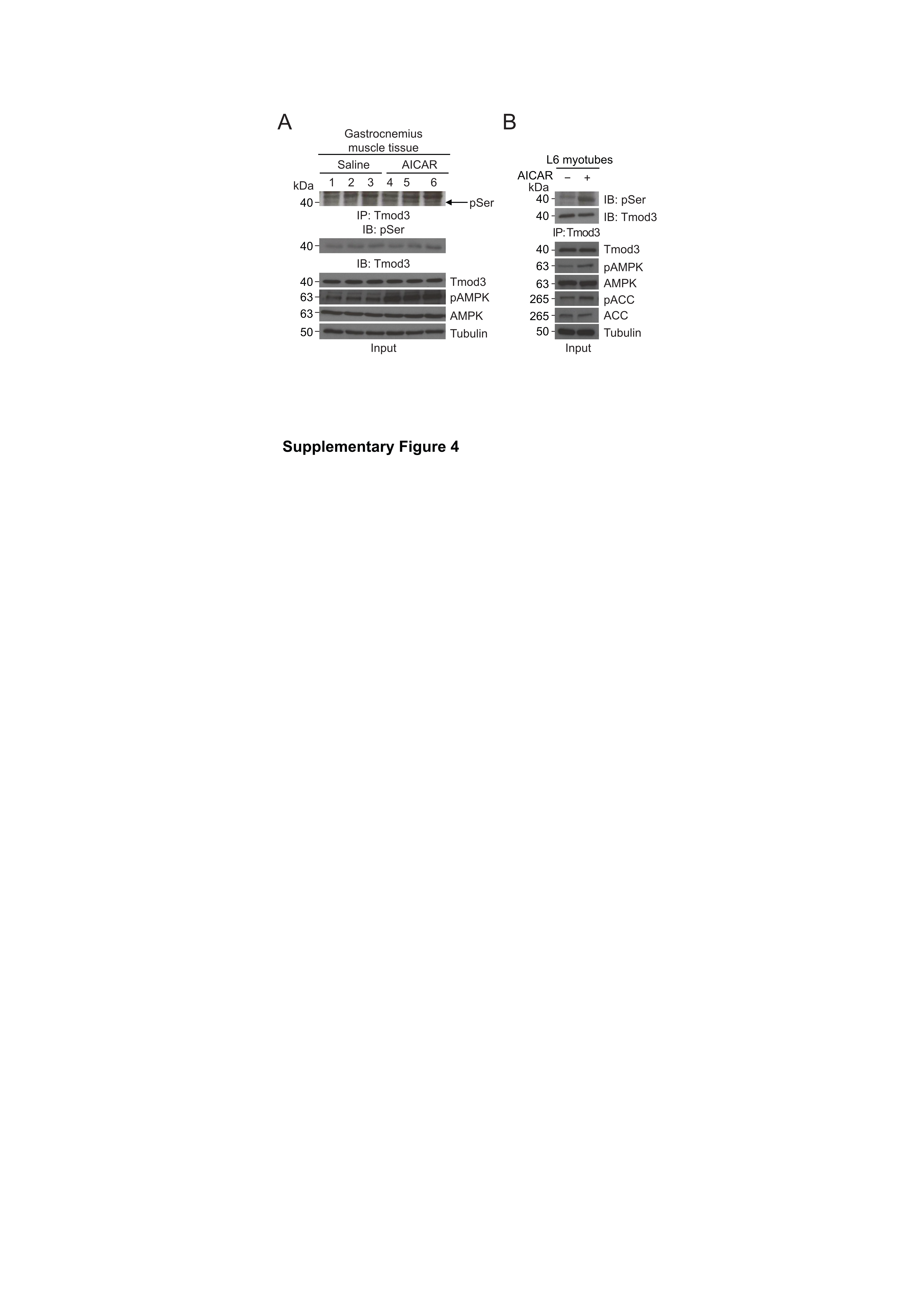

Supplement: Supplementary file 4 [file Image_4.tiff]

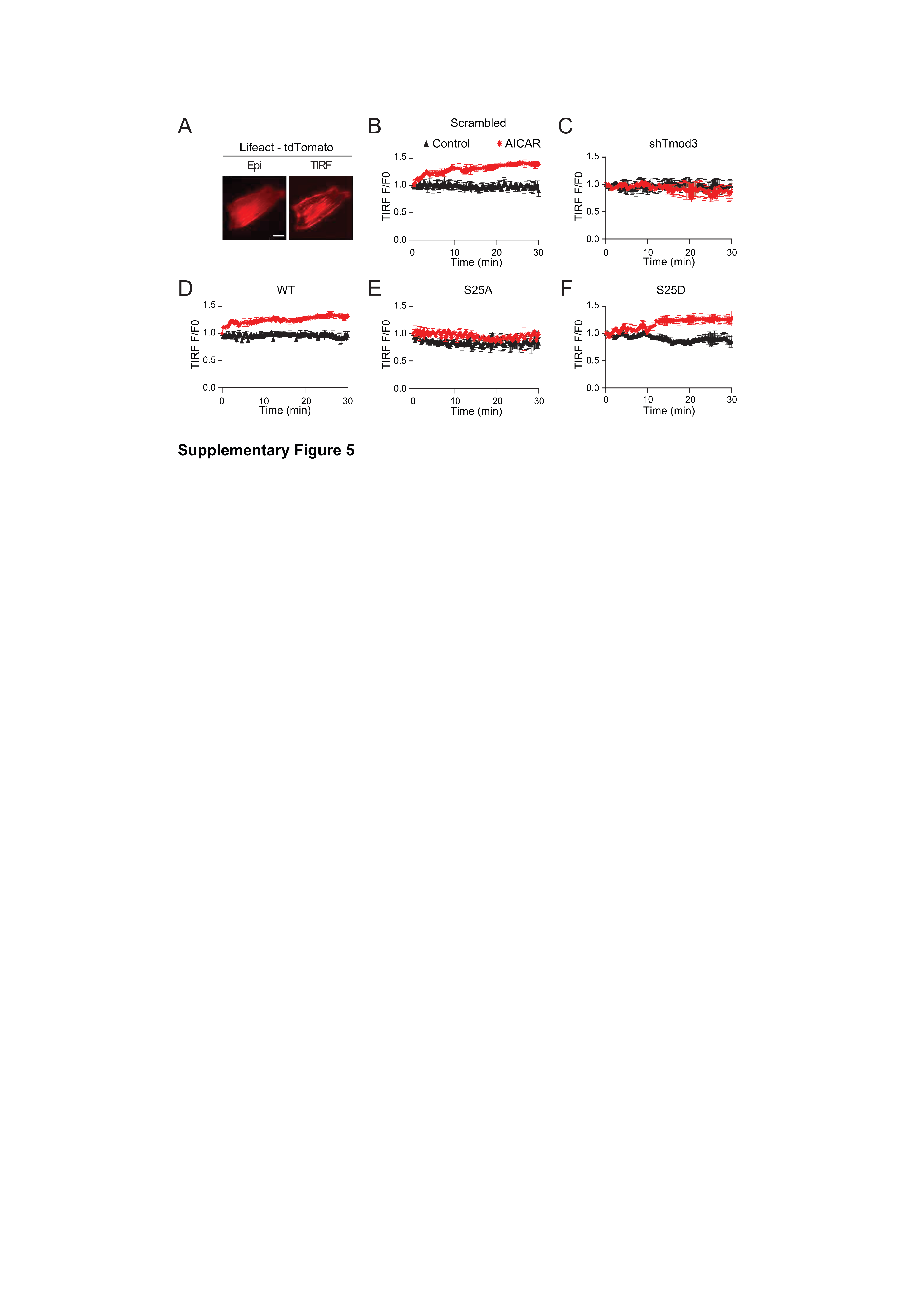

Supplement: Supplementary file 5 [file Image_5.tiff]
