# Supplementary material for: Tmod3 Phosphorylation Mediates AMPK-Dependent GLUT4 Plasma Membrane Insertion in Myoblasts
Source: Front Endocrinol (Lausanne). 2021 Apr 20;12:653557. doi: 10.3389/fendo.2021.653557 (PMC8095187; doi:10.3389/fendo.2021.653557)
Supplement: Supplementary file 6 [file Table_1.docx]

**Supplementary Materials**

**Supplementary Table 1: List of antibodies used in the study**

| **Antibodies** | **Isotype** | **Application** | **Dilution** | **Source/ Catalogue No.** |
| --- | --- | --- | --- | --- |
| Anti-FLAG-M2  Affinity Gel | Mouse | IP | 15µl/2mg protein | Sigma/A2220 |
| Anti-FLAG | Mouse | WB | 1:1000 | Sigma/F1804 |
| Anti-Tubulin | Mouse | WB | 1.:4000 | Sigma/T5168 |
| Anti-AMPK | Rabbit | WB | 1:1000 | Cell signaling/  2532S |
| Anti-phospho-  AMPK-Thr-172 | Rabbit | WB | 1:1000 | Cell signaling/  2535L |
| Anti-Acetyl CoA  Carboxylase | Rabbit | WB | 1:1000 | Cell signaling/  3662S |
| Anti-p-Acetyl CoA Carboxylase (S79) | Rabbit | WB | 1:1000 | Cell signaling/  3661S |
| Anti-phospho-  Serine | Rabbit | WB  IP | 1:1000  1µl/1mg protein | Life Technologies  (Invitrogen)/  61-8100 |
| Anti-Myc,  clone 9E10 | Mouse | WB  IF | 1:1000  1:400 | Santa Cruz/  SC-40 |
| Anti-Tmod3 | Rabbit | WB | 1:1000 | Aviva/ARP 55078-P050 |
| Anti-GLUT4,  clone 1F8 | Mouse | WB/IP/IF | 1:1000 | R&D Systems |
| Anti-β actin | Mouse | WB  IF | 1:1000  1:400 | Santa Cruz/  SC-47778 |
| Anti-γTm9d | Mouse | WB/ Detection  of Tpm3.1 | 1:1000 | Peter W. Gunning |
| Anti-δTm9d | Rabbit | WB/ Detection  of Tpm4.2 | 1:1000 | Peter W. Gunning |
| Anti-CG1 | Mouse | WB/ Detection  of Tpm2.1 | 1:1000 | Peter W. Gunning |
| Anti-αTm9d | Mouse | WB/ Detection  of Tpm2.1, 1.6, 1.7, 1.8, 1.9&1.4 | 1:1000 | Peter W. Gunning |

**Supplementary Table 2: List of plasmids used in the study**

| **Plasmids** | **Sense / Antisense primers** | **Remarks** |
| --- | --- | --- |
| pCMV5-FLAG-mTmod3-WT | 5’-GGA ATT CAG ATG GCA CTG CCG TTC CGG AAG  3’-CGG GAT CCC GTT ACT GGT GGT CTC CTT CAA TTC G | 5’–EcoRI;  3’–BamHI Mouse 3T3-L1 cDNA library |
| pCMV5-FLAG-mTmod3-S25A | 5’-CTT GGC AAG CTG GCC GAA TCA GAG C  3’-GCT CTG ATT CGG CCA GCT TGC CAA G | SDM phospho-defective mutant |
| pCMV5-FLAG-mTmod3-S25D | 5’-CTT GGC AAG CTG GAC GAA TCA GAG C  3’-GCT CTG ATT CGT CCA GCT TGC CAA G | SDM phospho-mimetic mutant |
| pCMV5-FLAG-mTmod3-S27A | 5’-GCT GTC CGA AGC AGA GCT GAA ACA G  3’-CTG TTT CAG CTC TGC TTC GGA CAG C | SDM phospho-defective mutant |
| pCMV5-FLAG-mTmod3-S25/27A | 5’-CTT GGC AAG CTG GCC GAA GCA GAG CTG AAA C  3’-GTT TCA GCT CTG CTT CGG CCA GCT TGC CAA G | SDM phospho-defective mutant |
| pCMV5-FLAG-mTmod3-L29G | 5’-CTG TCC GAA TCA GAG GGG AAA CAG CTG GAG AC  3’-GTC TCC AGC TGT TTC CCC TCT GAT TCG GAC AG | SDM defective in Tropomyosin-binding |
| pCMV5-FLAG-mTmod3-L134D | 5’-CAG AGC TGT GCG ACG ATG CAG CTA TTC TTG G  3’-CCA AGA ATA GCT GCA TCG TCG CAC AGC TCT G | SDM defective in Tropomyosin-binding |
| pCMV5-FLAG-mTmod3-LL | 5’-CTG TCC GAA TCA GAG GGG AAA CAG CTG GAG AC  3’-GTC TCC AGC TGT TTC CCC TCT GAT TCG GAC AG  5’-CAG AGC TGT GCG ACG ATG CAG CTA TTC TTG G  3’-CCA AGA ATA GCT GCA TCG TCG CAC AGC TCT G | SDM defective in Tropomyosin-binding |
| pCMV5-FLAG-mTmod3-RR | 5’-GGG AAA ACG ATG CTC ATC TTG TTG AAG  3’-CTT CAA CAA GAT GAG CAT CGT TTT CCC | SDM resistance to  pLKO.1-shTmod3 |
| pCMV5-FLAG-m-AMPK-WT |  | Addgene Plasmid #15991 |
| pCMV5-FLAG-m-AMPK-CA | 5’-GTG ATG AAC AGT TTA TAG TAC AGT GGT GAC CC  3’-GGG TCA CCA CTG TAC TAT AAA CTG TTC ATC AC |  |
| pCMV5-FLAG-m-AMPK-DN |  | Addgene Plasmid  #15992 |
| pLenti-FLAG-mTmod3-series | 5’-CGC GCT AGC GCC ATG GAC TAC AAG GAC GAT  3’-CGG TTA ACG TTA CTG GTG GTC TCC TTC AAT TCG |  |
| pLKO.1-shTmod3 | 5’-CCG GCC CTT GAT CCA GAG TTA GAA GCT CGA GCT TCT AAC TCT GGA TCA AGG GTT TTT G  3’-GGG AAC TAG GTC TCA ATC TTC GAG CTC GAA GAT TGA GAC CTA GTT CCC AAA AAC TTA A | shRNA against rat Tmod3 |
| pLKO.1-scrambled | 5’-CCG GAC AAC AGC CAC AAC GTC TAT ACT CGA GTA TAG ACG TTG TGG CTG TTG TTT TTT G  3’-AAT TCA AAA AAC AAC AGC CAC AAC GTC ATA CTC GAG TAT AGA CGT TGT GGC TGT TGT | shRNA against GFP |
| pMyc-GLUT4-mCherry | 5’-CGC GAA GCC GAA GAA CAG AAA CTG ATC TCT GAA GAA GAC CTG CTG AAG  3’-CGC GCT TCA GCA GGT CTT CTT CAG AGA TCA GTT TCT GTT CTT CGG CTT | Annealing oligos carrying Myc sequence; MluI site insertion into sequence encoding 1^st^ exofacial loop of pGLUT4-mCherry |
| pLifeact-Tdtomato | 5’–ATT CAT GGG CGT GGC CGA CCT GAT CAA GAA GTT CGA GAG CAT CAG CAA GGA GGA G  3’-CCG GCT CCT CCT TGC TGA TGC TCT CGA ACT TCT TGA TCA GGT CGG CCA CGC CCA TG | Annealing oligos carrying Lifeact sequence – EcoRI + AgeI insertion into pTdtomato-N1 (*49*) |
| pLenti-Lifeact-Tdtomato |  | pLifeact-Tdtomato-N1-NheI + HpaI cut, ligate into pLenti-hiko vector NheI + HpaI |
| pGEX-KG-mTmod3-series | 5’-GGA ATT CAG ATG GCA CTG CCG TTC CGG AAG  3’-CGG TTA ACG TTA CTG GTG GTC TCC TTC AAT TCG | PCR→ EcoRI + HpaI cut, ligate into pGEX-KG EcoRI + (XhoI) |

**Supplementary Table 3: List of primers used in the study**

| **Target Gene (Rat)** | **Sense primers** | **Antisense primers** |
| --- | --- | --- |
| Tmod1 | \| 5’-TGAGCTAGACCCTGATAATGCAC-3’ \| \| --- \| | 5’-CGGTCCTTAAATTCCTTCGCTTG-3’ |
| Tmod2 | \| 5’-GGCCGGTGAGGAATGTCGTC-3’ \| \| --- \| \|  \| | 5’-TCCTTCAGGGTTGGAATTGGA-3’ |
| Tmod3 | \| 5’-GAGACTATAAGGACCTGGATGAG-3’ \| \| --- \| \|  \| | 5’-CCTGTGGCGGACTTTGATGT-3’ |
| Tmod4 | \| 5’-CTGGACTGAGACAACGTGAC-3’ \| \| --- \| \|  \| | 5’-TCTTTGACTTCTAGCGCCTGT-3’ |
| GAPDH | 5’-GGCAAGTTCAACGGCACAG-3’ | 5’-CGCCAGTAGACTCCACGAC-3’ |
| Myf5 | \| 5’-CTCCCTCTCTGCTGAAACCA-3’ \| \| --- \| \|  \| | 5’-CGACTCTTGGCTCAAACTGG-3’ |
| Myf6 | \| 5’-GTACCCTATCCCCTTGCCAG-3’ \| \| --- \| \|  \| | 5’-CTGCTTTCCGACGATCTGTG-3’ |
| Myogenin | 5’-CATCCAGTACATTGAGCGCC-3’ | 5’-GCGAGCAAATGATCTCCTGG-3’ |
| GLUT4 | 5’-GCCGGACATTTGACCAGATC-3’ | 5’-GAGGTAAGGGAAGAGAGGGC-3’ |
| MyoD1 | \| 5’-CCGCTACATTGAAGGTCTGC-3’ \| \| --- \| \|  \| | 5’-TCCATCATGCCATCAGAGCA-3’ |

**Supplementary Methods**

**Mouse Work**

Mice (C57BL6/J, male, 5 – 7 weeks old) used in this study were maintained in animal facility of Biological Resource Centre, A*STAR. All the animal experiments were conducted in accordance with the regulations of Institutional Animal Care and Use Committee (IACUC #161122). The mice were injected i.p. with 250 mg/kg AICAR in sterile 0.9% NaCl or with 0.9% NaCl as previously described (78) and after 6 hours of treatment the gastrocnemius muscle was harvested for RNA and protein analysis.

**Cell Culture**

L6 myoblasts (ATCC) were cultured and differentiated into myotubes according to standard protocols (79). Briefly, L6 myoblasts were cultured in α-MEM supplemented with 10% heat inactivated fetal bovine serum and 1% penicillin-streptomycin (10,000 U/mL) at 37°C and 5% CO_2_. 4X10^4^/mL cells were seeded in 12-well plates and two days later after nearly 70% confluence, cells were washed with warm PBS twice and switched to differentiation medium containing 2% fetal bovine serum and 17.8 mM NaHCO_3_ for 4 days. The medium was changed every 24 hours.

### Gene expression analysis by RT-qPCR

Total RNA from L6 myoblasts or myotubes was extracted using RNeasy Mini Kit (Cat. No: 74016, Qiagen). First-strand cDNA synthesis was performed using one μg of RNA after DNase I treatment (Applied Biosystems TaqMan Reverse Transcription Reagents). The samples were diluted and cDNA was subjected to RT-qPCR using SYBR Green dyes along with appropriate primers. 2^−ΔΔ^*^C^*^t^ method was used to calculate the relative expression of mRNA abundance. Gene expression was normalized to GAPDH and compared with control group. Primers used for analysis are listed in Supplementary Table 3.

**Statistical Analysis**

Colocalisation analysis was performed using JACoP Plugin in ImageJ as previously described (80). All statistical analysis were performed using Prism 7.0 (GraphPad, San Diego, CA). All data are presented as mean values ± SEM for designated number of experiments. Significance of difference was tested by Student’s t-test.

**Figure Legends**

**Supplementary Figure 1 |** AMPK regulates GLUT4 translocation to the PM and glucose uptake in L6 myoblasts. **(A)** L6 myotubes were serum starved for 2 hours, treated with DMSO as control, insulin (100 nM) with and without Wortmannin (200 nM) for 20 minutes, and AICAR (2 mM) with and without Compound C (10 µM) for 30 minutes for [^3^H]-2-Deoxyglucose uptake assay. Data are presented as mean ± SEM of three independent experiments (ANOVA with Dunnett’s multiple comparison test). ^*^p<0.05. **(B)** Expression of different Tmod isoforms in L6 myoblasts by RT-qPCR. GAPDH was used as internal control. Data are presented as mean ± SEM of three independent experiments. ^****^p<0.0001. **(C)** Expression of Tmod3, GLUT4 and different myogenic differentiation markers in L6 myoblasts (D0) and differentiated L6 myotubes at day 6 (D6) by RT-qPCR. GAPDH was used as internal control. Data are presented as mean ± SEM of five independent experiments. ^****^p<0.0001. **(D)** Knockdown of Tmod3 expression. L6 myoblasts were transduced with lenti virus of shRNA targeting rat Tmod3 or control shRNA targeting GFP (Scramble). Forty-eight hours after transduction, mRNA levels of Tmod isoforms were analyzed by RT-qPCR. GAPDH was used as internal control. Data are presented as mean ± SEM of five independent experiments. ^***^p<0.001 and ^****^p<0.0001.

**Supplementary Figure 2 |** Tmod3 is associated with cortical F-actin and GLUT4 in L6 myoblasts. **(A)** L6 myoblasts stably expressing FLAG-Tmod3 were serum starved for 2 hours and treated with or without AICAR (2 mM) for 30 minutes. The cells were fixed and permeabilized with 0.1% Triton X-100 in PBS before immuno-staining with mouse anti-FLAG and rabbit anti-Actin antibody followed by Alexa Fluor-488 conjugated goat anti-mouse and Alexa Fluor-568-Phalloidin conjugated goat anti-rabbit secondary antibodies, respectively. **(B)** Pearson’s correlation coefficient of Tmod3 and Actin in L6 myoblast under control and AICAR stimulation. Data are representative of 10 images from three independent experiments. **(C)** Mander’s overlapping coefficient of Tmod3 and Actin in L6 myoblast under control and AICAR stimulation. Data are representative of 10 images from three independent experiments. **(D)** Co-localization of FLAG-Tmod3 and Myc-GLUT4-mCherry in response to AICAR stimulation. L6 myoblasts stably expressing FLAG-Tmod3 and Myc-GLUT4-mcherry were serum starved for 2 hours and treated with or without AICAR (2 mM) for 30 minutes. Myoblasts were fixed and permeabilized with 0.1% Triton X-100 in PBS before immuno-staining with mouse anti-FLAG antibody followed by Alexa Fluor-488 conjugated goat anti-mouse. The samples were subjected to confocal microscopic imaging. **(E)** Pearson’s correlation coefficient of Tmod3 and GLUT4 in L6 myoblast under control and AICAR stimulation. Data are representative of 10 images from three independent experiments. **(F)** Mander’s overlapping coefficient of Tmod3 and GLUT4 in L6 myoblast under control and AICAR stimulation. Data are representative of 10 images from three independent experiments.

**Supplementary Figure 3 |** Tmod3 co-localizes with phospho-AMPK in L6 myoblasts. **(A)** L6 myoblasts stably expressing FLAG-Tmod3 were serum starved for 2 hours and treated with or without AICAR (2 mM) for 30 minutes. The cells were fixed and permeabilized with 0.1% TritonX-100 in PBS before immuno-staining with mouse anti-FLAG and rabbit anti-phospho-AMPK (Thr-172) antibody followed by Alexa Fluor-488 conjugated goat anti-mouse and Alexa Fluor-568-Phalloidin conjugated goat anti-rabbit secondary antibodies respectively. **(B)** Pearson’s correlation coefficient of Tmod3 and pAMPK in L6 myoblast under control and AICAR stimulation. Data are representative of 10 images from three independent experiments. ^****^p<0.0001. **(C)** Mander’s overlapping coefficient of Tmod3 and pAMPK in L6 myoblast under control and AICAR stimulation. Data are representative of 10 images from three independent experiments. ^****^p<0.0001. **(D)** Consistent phospho-AMPK signals in both scrambled and Tmod3-KD L6 myoblasts under AICAR stimulation. The samples were subjected to confocal microscopic imaging. Scale bar: 10 μm. Fluorescence intensities were quantified along the lines as shown in the figures. Data are representative of 3 independent experiments. **(E)** Pearson’s correlation coefficient of Tmod3 and pAMPK in Scrambled and shTmod3-L6 myoblast under control and AICAR stimulation. Data are representative of 10 images from three independent experiments (ANOVA with Dunnett’s multiple comparison test). ^****^p<0.0001. **(F)** Mander’s overlapping coefficient of Tmod3 and pAMPK in Scrambled and shTmod3-L6 myoblast under control and AICAR stimulation. Data are representative of 10 images from three independent experiments (ANOVA with Dunnett’s multiple comparison test). ^****^p<0.0001.

**Supplementary Figure 4 |** Tmod3 is a novel AMPK substrate. **(A)** AICAR induces Tmod3 phosphorylation *in vivo*. After overnight fasting, 5 – 7 weeks old wild-type C57BL/6 mice were subjected to intraperitoneal injection with either saline (0.9% NaCl) or AICAR (250 mg/kg body weight). After 6 hours, the mice were sacrificed and gastrocnemius muscle tissues were extracted. Lysates from gastrocnemius muscles were immuno-precipitated with anti-Tmod3 antibody and phosphorylation of Tmod3 was detected with anti-Phospho Serine antibody. Data are representative of two independent experiments. **(B)** Lysates from L6 myotubes treated with or without AICAR (2 mM) were immuno-precipitated with anti-Tmod3 antibody and phosphorylation of Tmod3 was detected with anti-Phospho Serine antibody. Data are representative of three independent experiments.

**Supplementary Figure 5 |** Representative examples of TIRF-Lifeact-tdTomato F-actin remodeling analysis. **(A)** L6 myoblasts stably expressing Lifeact-tdTomato under TIRF microscope. **(B-F)** Measurement on the peripheral regions after removal of background fluorescence, TIRF intensities of ROIs measured over time were normalized to the intensity measured at zero time point, averaged and plotted against the time to indicate the time course of F-actin remodeling. N = 8-10 cells per condition were analyzed.

**Supplementary Movies**

**Supplementary Movie 1A | Time-lapse images of AMPK-induced actin remodeling captured under TIRFM. Timelapse images were recorded for 30 min at an interval of 15 seconds**

Scr-Control: L6 myoblast expressing Lifeact-tdTomato was imaged using TIRFM at the basal state. Scale bar: 10 μm. Representative ROI has been marked with a white square box.

**Supplementary Movie 1B | Time-lapse images of AMPK-** **induced actin remodeling captured under TIRFM. Timelapse images were recorded for 30 min at an interval of 15 seconds**

Scr-AICAR: L6 myoblast expressing Lifeact-tdTomato was imaged using TIRFM under 2 mM AICAR stimulation. Scale bar: 10 μm. Representative ROI has been marked with a white square box.

**Supplementary Movie 1C | Time-lapse images of AMPK-** **induced actin remodeling captured under TIRFM. Timelapse images were recorded for 30 min at an interval of 15 seconds**

shTmod3-Control: L6 myoblast with Tmod3 knockdown and expressing Lifeact-tdTomato was imaged using TIRFM at the basal state. Scale bar: 10 μm. Representative ROI has been marked with a white square box.

**Supplementary Movie 1D | Time-lapse images of AMPK-** **induced actin remodeling captured under TIRFM. Timelapse images were recorded for 30 min at an interval of 15 seconds**

shTmod3-AICAR: L6 myoblast with Tmod3 knockdown and expressing Lifeact-tdTomato was imaged using TIRFM under 2 mM AICAR stimulation. Scale bar: 10 μm. Representative ROI has been marked with a white square box.

**Supplementary Movie 2A | Time-lapse images of AMPK-** **induced actin remodeling in cells upon knockdown and re-expression of Tmod3-WT recorded under TIRFM. Time-lapse images were recorded for 30 min at an interval of 15 seconds**

shTmod3+Tmod3-WT-Control: Tmod3-WT and Lifeact-tdTomato stably expressing cell was imaged using TIRFM at the basal state. Scale bar: 10 μm. Representative ROI has been marked with a white square box.

**Supplementary Movie 2B | Time-lapse images of AMPK-** **induced actin remodeling in cells upon knockdown and re-expression of Tmod3-WT recorded under TIRFM. Time-lapse images were recorded for 30 min at an interval of 15 seconds**

shTmod3+Tmod3-WT-AICAR: Tmod3-WT and Lifeact-tdTomato stably expressing cell was imaged using TIRFM under 2 mM AICAR stimulation. Scale bar: 10 μm. Representative ROI has been marked with a white square box.

**Supplementary Movie 2C | Time-lapse images of AMPK-** **induced actin remodeling in cells upon knockdown and re-expression of Tmod3-S25A recorded under TIRFM. Time-lapse images were recorded for 30 min at an interval of 15 seconds**

shTmod3+Tmod3-S25A-Control: Tmod3-S25A and Lifeact-tdTomato stably expressing cell was imaged using TIRFM at the basal state. Scale bar: 10 μm. Representative ROI has been marked with a white square box.

**Supplementary Movie 2D | Time-lapse images of AMPK-** **induced actin remodeling in cells upon knockdown and re-expression of Tmod3-S25A recorded under TIRFM. Time-lapse images were recorded for 30 min at an interval of 15 seconds**

shTmod3+Tmod3-S25A-AICAR: Tmod3-S25A and Lifeact-tdTomato stably expressing cell was imaged using TIRFM under 2 mM AICAR stimulation. Scale bar: 10 μm. Representative ROI has been marked with a white square box.

**Supplementary Movie 2E | Time-lapse images of AMPK-** **induced actin remodeling in cells upon knockdown and re-expression of Tmod3-S25D recorded under TIRFM. Time-lapse images were recorded for 30 min at an interval of 15 seconds**

shTmod3+Tmod3-S25D-Control: Tmod3-S25D and Lifeact-tdTomato stably expressing cell was imaged using TIRFM at the basal state. Scale bar: 10 μm. Representative ROI has been marked with a white square box.

**Supplementary Movie 2F| Time-lapse images of AMPK-** **induced actin remodeling in cells upon knockdown and re-expression of Tmod3-S25D recorded under TIRFM. Time-lapse images were recorded for 30 min at an interval of 15 seconds**

shTmod3+Tmod3-S25D-AICAR: Tmod3-S25D and Lifeact-tdTomato stably expressing cell was imaged using TIRFM under 2 mM AICAR stimulation. Scale bar: 10 μm. Representative ROI has been marked with a white square box.

**Reference**

78 Jager S, Handschin C, St-Pierre J, Spiegelman BM. AMP-activated protein kinase (AMPK) action in skeletal muscle via direct phosphorylation of PGC-1alpha. *Proc Natl Acad Sci U S A* (2007) 104:12017-12022, doi:10.1073/pnas.0705070104

79 Lawson MA, Purslow PP. Differentiation of myoblasts in serum-free media: effects of modified media are cell line-specific. *Cells Tissues Organs* (2000) 167:130-137, doi:10.1159/000016776

80 Bolte S, Cordelières FP. A guided tour into subcellular colocalization analysis in light microscopy. *J Micros* (2006) 224:213-232, doi:10.1111/j.1365-2818.2006.01706.x

**Supplemental Information**

Supplemental Information can be found with this paper online at

<https://www.frontiersin.org/articles/10.3389/fendo.2021.653557/full#supplementary-material>
